# Cigarette Smoke Affects Keratinocytes SRB1 Expression and Localization via H_2_O_2_ Production and HNE Protein Adducts Formation

**DOI:** 10.1371/journal.pone.0033592

**Published:** 2012-03-19

**Authors:** Claudia Sticozzi, Giuseppe Belmonte, Alessandra Pecorelli, Beatrice Arezzini, Concetta Gardi, Emanuela Maioli, Clelia Miracco, Marzia Toscano, Henry Jay Forman, Giuseppe Valacchi

**Affiliations:** 1 Department of Mechanical and Structural Engineering, University of Trento, Trento, Italy; 2 Department of Biomedical Sciences, University of Siena, Siena, Italy; 3 Department of Pathophysiology, Experimental Medicine and Public Heath, University of Siena, Siena, Italy; 4 Department of Physiology, University of Siena, Siena, Italy; 5 Department of Human Pathology and Oncology, University of Siena, Siena, Italy; 6 Davis School of Gerontology, University of Southern California, Los Angeles, California, United States of America; 7 School of Natural Sciences, University of California at Merced, Merced, California, United States of America; 8 Department of Evolutionary Biology, University of Ferrara, Ferrara, Italy; 9 Department of Food and Nutrition, Kyung Hee University, Seoul, Korea; Kaohsiung Chang Gung Memorial Hospital, Taiwan

## Abstract

Scavenger Receptor B1 (SR-B1), also known as HDL receptor, is involved in cellular cholesterol uptake. Stratum corneum (SC), the outermost layer of the skin, is composed of more than 25% cholesterol. Several reports support the view that alteration of SC lipid composition may be the cause of impaired barrier function which gives rise to several skin diseases. For this reason the regulation of the genes involved in cholesterol uptake is of extreme significance for skin health. Being the first shield against external insults, the skin is exposed to several noxious substances and among these is cigarette smoke (CS), which has been recently associated with various skin pathologies. In this study we first have shown the presence of SR-B1 in murine and human skin tissue and then by using immunoblotting, immunoprecipitation, RT-PCR, and confocal microscopy we have demonstrated the translocation and the subsequent lost of SR-B1 in human keratinocytes (cell culture model) after CS exposure is driven by hydrogen peroxide (H_2_O_2_) that derives not only from the CS gas phase but mainly from the activation of cellular NADPH oxidase (NOX). This effect was reversed when the cells were pretreated with NOX inhibitors or catalase. Furthermore, CS caused the formation of SR-B1-aldheydes adducts (acrolein and 4-hydroxy-2-nonenal) and the increase of its ubiquitination, which could be one of the causes of SR-B1 loss. In conclusion, exposure to CS, through the production of H_2_O_2_, induced post-translational modifications of SR-B1 with the consequence lost of the receptor and this may contribute to the skin physiology alteration as a consequence of the variation of cholesterol uptake.

## Introduction

The scavenger receptor class B type I (SR-BI), a transmembrane protein, mediates selective lipid uptake from hydrophobic lipoprotein cores [1], [2], and facilitates the cellular uptake of cholesterol (mainly cholesteryl esters) by intervening in the binding of the lipoprotein with the outer surfaces of cells, through a process in which the cholesterol esters are internalized without the net internalization and degradation of the lipoprotein itself although Silver et al. have shown that in polarized liver cells SR-B1 is able to facilitate uptake of the whole HDL particle via a transcytosis process [3].

One of the tissues where cholesterol is of great importance is certainly the skin. The skin consists of two main layers, the inner dermis, mainly composed of fibroblasts, vessels and nerves and connective tissue matrix, and the outer epidermis, which contains mainly keratinocytes. These cells progressively differentiate into enucleate corneocytes, which are imbedded in a lipid matrix rich in ceramides, cholesterol and free fatty acids forming the stratum corneum (SC) (the outermost part of the epidermis).

Cholesterol represents about one-quarter of the lipid content of the SC. It is an essential component of all cell membranes. Cholesterol is implicated in corneocyte desquamation and cohesion and keratinocyte differentiation. Cholesterol is also required in keratinocytes to form lamellar bodies. Secretion of lamellar bodies then delivers lipids, including cholesterol, to the extracellular spaces of the SC, which mediate permeability barrier function [4]. The ability to limit the transcutaneous movement of water and electrolytes is required for terrestrial life. Although the cholesterol synthesis rate is high under basal conditions, cholesterol synthesis increases after permeability barrier disruption [5], as do the levels of receptors that enhance the uptake of cholesterol into the cell including the LDL receptor and scavenger receptor class B type I [6], [7]. Inhibition of cholesterol synthesis perturbs permeability barrier function [8], and a selective deficiency in cholesterologenesis largely accounts for the barrier abnormality in chronologically aged epidermis [9], [10].

Because of its critical location, the cutaneous tissue is the first barrier against environmental insults such as UV radiation, CS, diesel fuel exhaust, halogenated hydrocarbons, heavy metals and O_3_ [11]. The skin is protected against these oxidative stressors by an elaborate system of antioxidant substances including vitamin C, vitamin E, uric acid, and GSH, and enzymes including superoxide dismutase (SOD), glutathione peroxidase (GPX), and catalase (CAT) that are depleted or inactivated, respectively, after exposure to such stressors. The loss of the antioxidants correlates with an increase in lipid peroxidation.

In the last 10 years, it has been shown that CS and the oxidative compounds that derive from the combustion of cigarettes can affect the skin [12], [13]. Furthermore, several skin diseases such as melanoma [14], [15], psoriasis [16] and dermatitis [17] have now been associated with CS exposure. The pathological effects of cigarette smoke (CS) have been extensively documented. CS contains over 4,600 compounds in gaseous and particulate states that are able to induce oxidative stress to cells and its toxic effect is mainly due to the presence of oxidants, including H_2_O_2_ [18] and volatile electrophilic compounds including α,β-unsaturated aldehydes. Acrolein (ACR) and 4-hydroxy-2-nonenal (HNE), which are highly reactive and potentially toxic, can also be generated during inflammation as a consequence of lipid peroxidation [19], [20]. These aldehydes, as well as H_2_O_2_, are capable of affecting a variety of biochemical processes, including transcription factor activation and gene expression, production of inflammatory cytokines, respiratory burst activation, and cell death [21]–[23]. Recently, our group was able to demonstrate that CS exposure modulates genes involved in cholesterol trafficking such as SR-B1 and ABCA1 in lung tissue [24], [25] and ABCA1 in skin tissue [26].

As a continuation of our previous work, the current study explored the modulation of SR-B1 in keratinocytes after CS exposure. Our results show that in keratinocytes, CS decreased SR-B1 levels in an H_2_O_2_ (exogenous and endogenous)-dependent manner. This effect is a consequence of the formation of aldehydes-adducts and subsequent SR-B1 degradation mediated by ubiquitination.

## Methods

### Cell Culture and Treatments

HaCaT cells, (a cell line gift from Dr. F. Virgili), were grown in Dulbecco's modified Eagle's medium High Glucose (Lonza, Milan, Italy), supplemented with 10% FBS, 100 U/ml penicillin, 100 µg/mL streptomycin and 2 mM L-glutamine as previously described [27]. Cell suspension containing 10 or 1×10^5^ viable cells/ml were used. Cells were incubated at 37°C for 24 h in 95% air/5% CO_2_ until 80% confluency.

HaCaT cells were treated with either acrolein (ACR) (Aldrich, Milwaukee, WI) or 4-hydroxy-2-nonenal (HNE) (Calbiochem, La Jolla, CA) or glucose oxidase (GO; type II from Aspergillus niger, 15.5 U/g; Calbiochem, La Jolla, CA) or pretreated (2 h) with PEG-catalase (PEG-CAT) or diphenyleneiodonium chloride (DPI) or MG-132 (Calbiochem, La Jolla, CA) before CS exposure, and then resuspended in DMEM medium supplemented with 10% FBS. After treatments for various time periods, cells were collected by centrifugation for the several assays described below.

### CS Exposure

Prior to CS exposure of the the cells, media was aspirated and fresh serum-free medium was added. Cells were then exposed for 50 min to CS. Control cells were exposed to filtered air for the same duration (50 min) after changing media.

The time and the method of exposure were chosen based on our previous work [18], [25], [27]. Under our experimental conditions no significant differences in cell viability as measured by Trypan blue exclusion was detected between control (air) and CS treated cells (data not shown).

HaCaT cells were exposed to fresh CS in an exposure system that generated CS by burning one UK research cigarette (12 mg tar, 1.1 mg nicotine) using a vacuum pump to draw air through the burning cigarette and leading the smoke stream over the cell cultures as described previously by our group [25]. After the exposure (air or CS), fresh media supplemented with 10% FBS was added to the cells.

### Immunocytochemistry

HaCaT cells were grown on coverslips at a density of 1×10^5^ cell/ml, and after CS exposure fixed in 4% paraformaldehyde in PBS for 30 min at 4°C. Cells were permeabilized for 15 min at room temperature with PBS containing 1% BSA, 0.2% Triton X-100, and 0.02% sodium azide, then the coverslips were blocked in PBS containing 1% BSA, 0.2% Nonidet P-40 and 0.02% sodium azide at room temperature for 1 hr. Coverslips were then incubated for 1 hr with primary antibody, followed by 1 hr with secondary antibodies. Nuclei were stained with 1 µg/ml DAPI (Molecular Probes) for 1 min after removal of secondary antibodies. Coverslips were mounted onto glass slides using with anti-fade mounting medium 1,4 diazabicyclooctane in glycerine (DABCO) and examined by the Zeiss Axioplan2 light microscope equipped with epifluorescence at 40× magnification. Negative controls for the immunostaining experiments were performed by omitting primary antibodies. Images were acquired and analyzed with Axio Vision Release 4.6.3 software.

### Immunohistochemistry

Human and mouse skin tissue were immersion fixed in 10% NBF (neutral-buffered formalin) for 24 hours at room temperature. Sections (4 µm) were deparaffinized in xylene and rehydrated in alcohol gradients. After dewaxing, sections were incubated overnight at 4°C with anti-SRB1 (Novus Biologicals, Inc.; Littleton, CO). Then slides were washed three times with PBS and endogenous peroxidase was blocked with 3% hydrogen peroxide in absolute methyl alcohol for 30 minutes at room temperature. Finally, the slides were incubated with EnVision+ System-HRP (DAKO, Glostrup, Denmark) for 45 minutes at room temperature. The reaction products were stained with diaminobenzidine (DAB), counterstained with Mayer's Hematoxylin and after drying were mounted with Eukitt mounting medium.

The animal protocol n G030806 was approved by the Istitutional Laboratory Animal Care and Use Committee of the University of Siena, Italy. In addition the local ethics committees approved the use of human samples, and all patients provided a signed informed consent form.

### Western blot Analysis

Total cell lysates were extracted in solubilization buffer containing 50 mM Tris (pH 7.5), 150 mM NaCl, 10% glycerol, 1% Nonidet P-40, 1 mM EGTA, 0.1% SDS, 5 mM N-ethylmaleamide (Sigma-Aldrich Corp.), protease and phosphatase inhibitor cocktails (Sigma–Aldrich Corp.) as described before [25].

Cells were harvested by centrifugation and proteins concentration was determined by the method of Bradford (Biorad Protein assay, Milan, Italy). Samples of 60 µg protein in 3× loading buffer (65 mM Tris base, pH 7.4, 20% glycerol, 2% sodium dodecyl sulfate, 5% β-mercaptoethanol and 1% bromophenol blue) were boiled for 5 min, loaded onto 10% sodium dodecyl sulphate–polyacrylamide electrophoresis gels and separated by molecular size. The gels were then electro-blotted onto nitrocellulose membranes which were then blocked for 1 hr in Tris-buffered saline, pH 7.5, containing 0.5% Tween 20 and 5% milk. Membranes were incubated overnight at 4°C with the appropriate primary antibody: SR-B1 (Novus Biologicals, Inc.; Littleton, CO), β-actin (Cell Signalling; Celbio, Milan, Italy), HNE (Millipore Corporation, Billerica, MA, USA), acrolein (gift from Prof. Uchida), p47*^phox^* and p67*^phox^* (Millipore Corporation, Billerica, MA, USA). The membranes were then incubated with horseradish peroxidase-conjugated secondary antibody for 1 hr, and the bound antibodies were detected using chemiluminescence (BioRad, Milan, Italy).The blots were then stripped and re-probed with β-actin (1∶1000) as the loading control. Images of the bands were digitized and the densitometry of the bands were performed using Image-J software.

### SR-B1 cellular localization

To assess the SR-B1 translocation to the plasma membrane, cells were exposed to CS and then homogenized on ice in Tris-HCl buffer, pH 7.4, containing 1 mM EGTA, 1 mM EDTA, protease and phosphatase inhibitors. Cell lysates were then separated by centrifugation (100,000×*g*, 30 min, 4°C). The supernatant containing the cytosolic fraction in the pellet were solubilized in lysis buffer containing 1% Triton X-100 followed by homogenization with a 25-gauge needle. An equal amount of proteins were loaded on SDS 10% PAGE and then transferred to a nitrocellulose membrane. Western blotting was performed as described above.

### Immunogold labelling

After CS exposure, HaCaT cells (1×10^6^ cell/ml) were fixed in 4% paraformaldehyde - 0.5% glutaraldehyde in 0.1 M phosphate buffer (PB) for 2 h at 4°C. Cells were harvested by centrifugation and washed in PBS overnight at 4°C. Cells, after dehydration, were infiltrated with LR-White Resin Hard Grade (TAAB Laboratories, England, UK) - ethanol 70% (2∶1) for 30′ at 4°C and then with pure LR-White Resin overnight at 4°C. Then, cells were placed in gelatine capsules and polymerized in fresh resin at 60°C for 24 h. Ultrathin sections (60–90 nm) were cut on a ultramicrotome (Ultratome Nova-LKB Bromma) and collected on 200-mesh nickel grids. For the immunogold labelling procedure, grids containing sections were immersed in a series of droplets of each solution on a strip of Parafilm in a humid chamber. The grids were incubated in 0.05 M tris-buffered saline-Tween20 (TBST), pH 7.6, and free aldehyde binding was quench using 0.1 M Glycine in PB for 20′ at room temperature, and then non-specific protein binding was blocked using 5% Normal Goat Serum (NGS)- 1% BSA in TBST for 30′ room temperature. They were then incubated overnight at 4°C in anti-SRB1 (1∶100) in 2% NGS-TBST. After treatment with primary antibody, grids were washed in TBST and then incubated in goat anti-rabbit IgG conjugated to 10 nm Gold particles (BBInternational, Cardiff, UK) diluted 1∶100 in 2% NGS-TBST for 2 h at room temperature. The grids were washed again in TBST. The grids were post-fixed on droplets of 3% glutaraldehyde in 0.06 M cacodylate buffer for 5′ and then rinse in TBST and distilled water, stained in aqueous uranyl acetate and lead citrate. The grids were examined and photographed using a Philips CM10 transmission electron microscope. For negative control, grids containing sections were incubated in 2% NGS-TBST without the primary antibody.

### Protein carbonyls

Carbonyl groups in proteins were determined by OxyBlot (Chemicon, USA). Briefly, after derivatization of carbonyl groups to dinitrophenylhydrazone (DNP-hydrazone) by reaction with dinitrophenylhydrazine (DNPH), the DNP-derivatized protein samples were separated by polyacrylamide gel electrophoresis followed by Western blotting.

### Immunoprecipitation

Cell lysates containing 300 µg of protein were mixed with Dynabeads protein G and 2 µg of polyclonal antibody against SR-B1. Following immunoprecipitation of SR-B1, the presence of HNE or Ubiquitin adducts was determined, after which proteins were separated by SDS-PAGE, electrotransferred to nitrocellulose membranes and immunoblotted with a HNE or ubiquitin antibody.

### H_2_O_2_ Measurement

Measurement of H_2_O_2_ was performed as described by Vecchio et al. [28], according to the method of Mohanty and co-workers [29], by monitoring the horseradish peroxidase (HRP)-catalyzed oxidation of the probe N-acetyl-3,7- dihydroxyphenoxazine (A6550; Molecular Probes, Eugene, OR, USA), which becomes highly fluorescent only after oxidation by H_2_O_2_. Briefly, HaCaT cells were washed twice with PBS and the medium was replaced by Krebs-Ringer phosphate buffer (200 µl/well), pH 7.4, containing 20 mM HEPES, 130 mM NaCl, 1.2 mM Na phosphate, 5 mM KCl, 2 mM CaCl_2_, 1.2 mM MgSO_4_ and 1 g/l glucose. The probe A6550 and HRP were added at final concentration of 50 µM and 1 U/ml, respectively, and fluorescence was read by a Perkin-Elmer fluorescence plate reader (Ex. 560 nm; Em. 642 nm).

### Detection of mitochondrial superoxide production

Superoxide production was measured using the indicator MitoSOX Red (Invitrogen), a mitochondrion-specific hydroethidine-derivative fluorescent dye, according to the manufacturer's instructions. Briefly, for live cell imaging, HaCaT cells were allowed to adhere on glass coverslips. After CS exposure, media was removed and cells were loaded with Mitosox Red (Invitrogen) (5 µM) in Hanks' Balanced Salt Solution for 10 min at 37°C. Cells were then washed and imaged on a Zeiss Axioplan2 inverted fluorescence microscope using a Rhodamine filter (MitoSOX was excited at 515 nm, and emitted light was measured from 520–620 nm).

### Statistical Analysis

For each of the variables tested, two-way analysis of variance (ANOVA) was used. A significant effect was indicated by a *P*-value<0.05. Data are expressed as mean ± S.D. of triplicate determinations obtained in 5 separate experiments.

## Results

### SR-B1 is expressed in human and mouse skin

**Figure 1:**
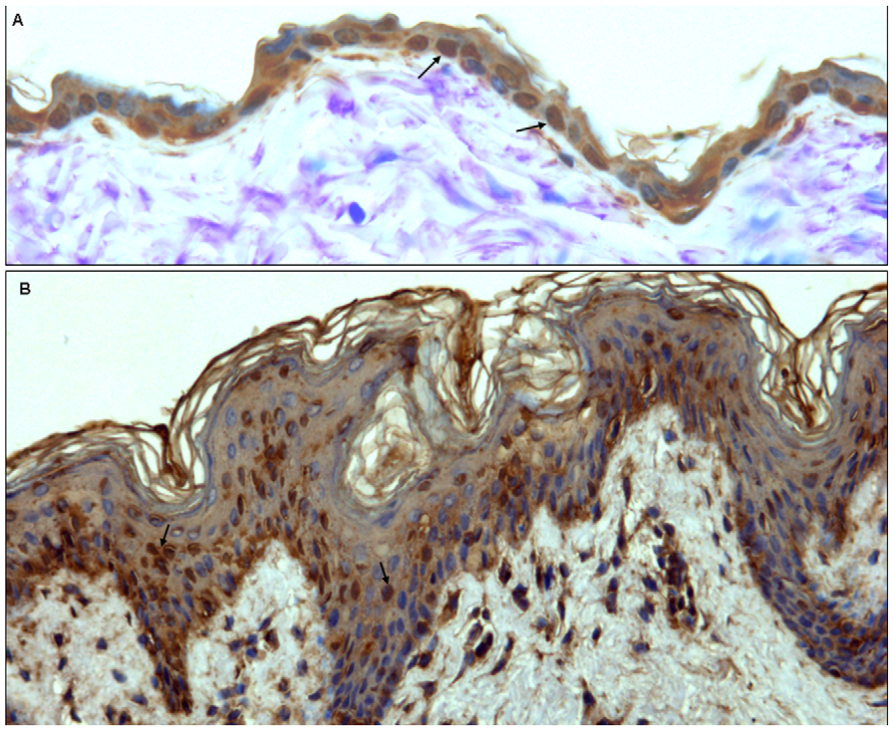
SR-B1 is expressed in epidermis of mouse (A) and human (B) skin. The anti SR-B1 antibody dekorates/stains most nuclei of keratinocytes (as the ones indicated by the arrows) in both murine and human normal skin SR-B1 immunohistochemistry, DAB, Original Magnification ×200 (human skin) and ×400 (mouse skin).

To evaluate the presence of SR-B1 in cutaneous tissue immunohistochemistry analysis was performed in samples from murine skin and from human biopsies. As shown in Fig. 1 a strong nuclear positivity to SR-B1 was demonstrated in normal keratinocytes of both murine (Fig. 1A) and human skin (Fig. 1B); in human skin, positivity was mainly detected in basal-suprabasal keratinocytes although also dermal fibroblasts showed an evident cytoplasmic positivity.

### CS exposure decreased SR-B1 levels

**Figure 2:**
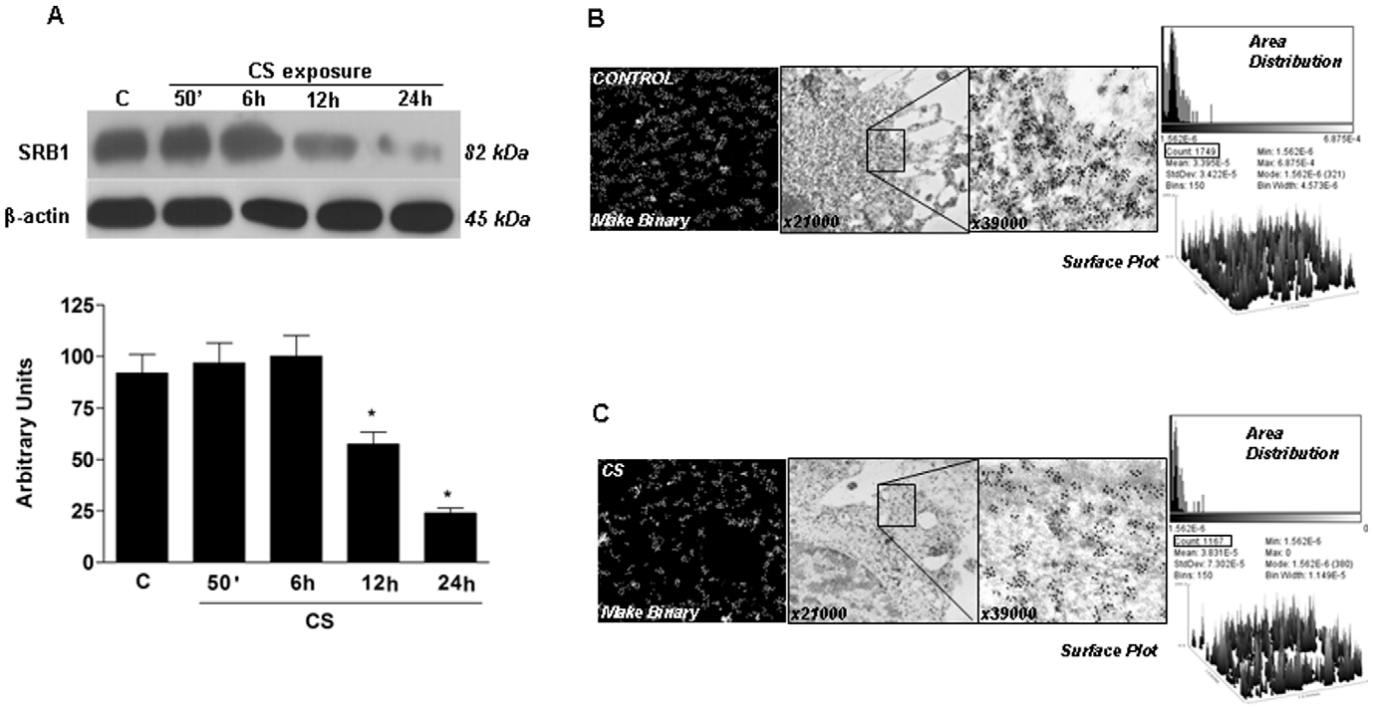
Exposure to CS decreased SR-B1 protein levels in HaCaT cells. Cells were exposed to CS for 50 min and cells were harvested at different time points (0–24 hrs). The Western blot shown in the top is representative of five experiments. Quantification of the SR-B1 bands is shown in the bottom panel. Data are expressed in arbitrary units (averages of five different experiments, *p<0.05). β-actin was used as loading control. Immunogold for SR-B1 confirm the decreased protein levels after CS exposure (B). IHC for SR-B1 is shown in the C panel (arrows).

We first assessed whether SR-B1 levels are modulated by CS exposure in HaCaT cells. As shown in Figure 2A, the protein levels of SR-B1 decreased markedly upon CS exposure starting at 12 h (2-fold) and reaching an almost 4-fold decrease 24 h after CS exposure. This effect was confirmed also by immunogold labelling analysis. As shown in Figure 2C, the protein levels of SR-B1 decreased markedly upon CS exposure compared with the air-exposed cells (Fig. 2B). This phenomena was not a consequence of cell viability modification (Trypan blue exclusion assay) as no significant differences were founded between control and treated cells (data not shown).

### CS exposure affects SR-B1 cellular localization and membrane protein levels

**Figure 3:**
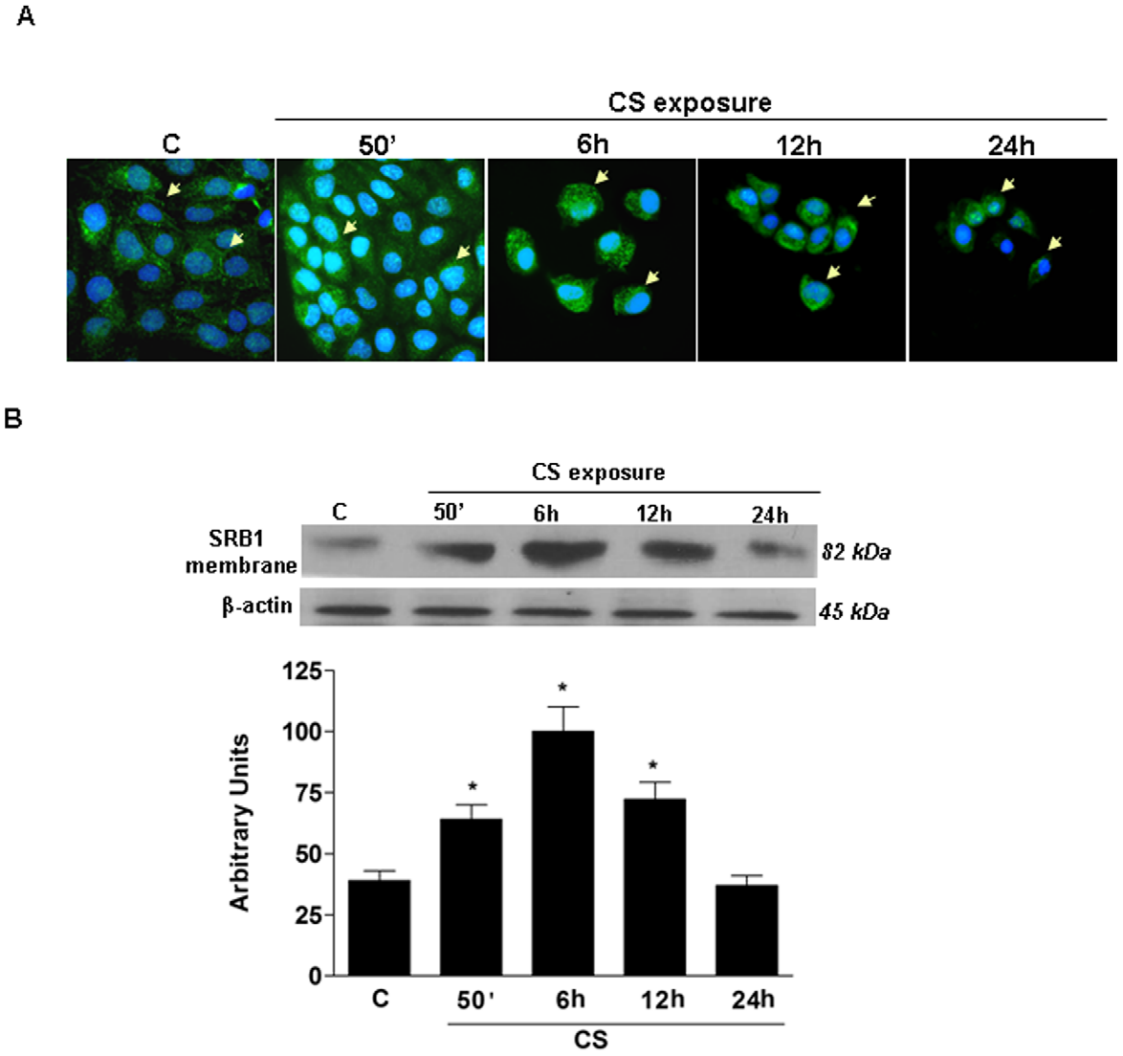
Cigarette Smoke exposure induces changes in SR-B1 levels and localization in human keratinocytes. Immunocytochemistry of HaCaT cells showing localization of SR-B1 (green) before and after CS exposure for different time points. Images are merged and are representative of at least 100 cells viewed in each experiments (n = 5). Nuclei (blue) were stained with DAPI. A) Cells overview at different time points 40×; B) Representative Western blot of proteins extracted from the membranes of cells exposed to Cigarette Smoke at different time points. The signals of SR-B1 protein levels were determined by densitometric analysis of the scanned images (bottom panel). Data are expressed in arbitrary units and are averages of the values for five different experiments. (*p<0.05).

Immunocytochemistry (ICC) showed that CS exposure caused the translocation of SR-B1 from the perinuclear area to the cell membrane resulting in an increase of SR-B1 membrane location. This pattern changed at 6 and 12 h post CS exposure as the perinuclear staining was almost completely lost and membrane staining was also decreased (Fig. 3). As shown in Fig. 3A, at 24 h SR-B1 levels were dramatically decreased in all cellular compartments and membranes. To quantify and confirm the movement and subsequent loss of SR-B1, we performed an immunoblot analysis on the cell membrane protein extract. Fig. 3B showed the increase in SR-B1 membrane levels (circa 3 fold after 6 hr) and the following significant decrease at the later time points.

### CS exposure induced Michael addition of aldehydes to proteins

**Figure 4:**
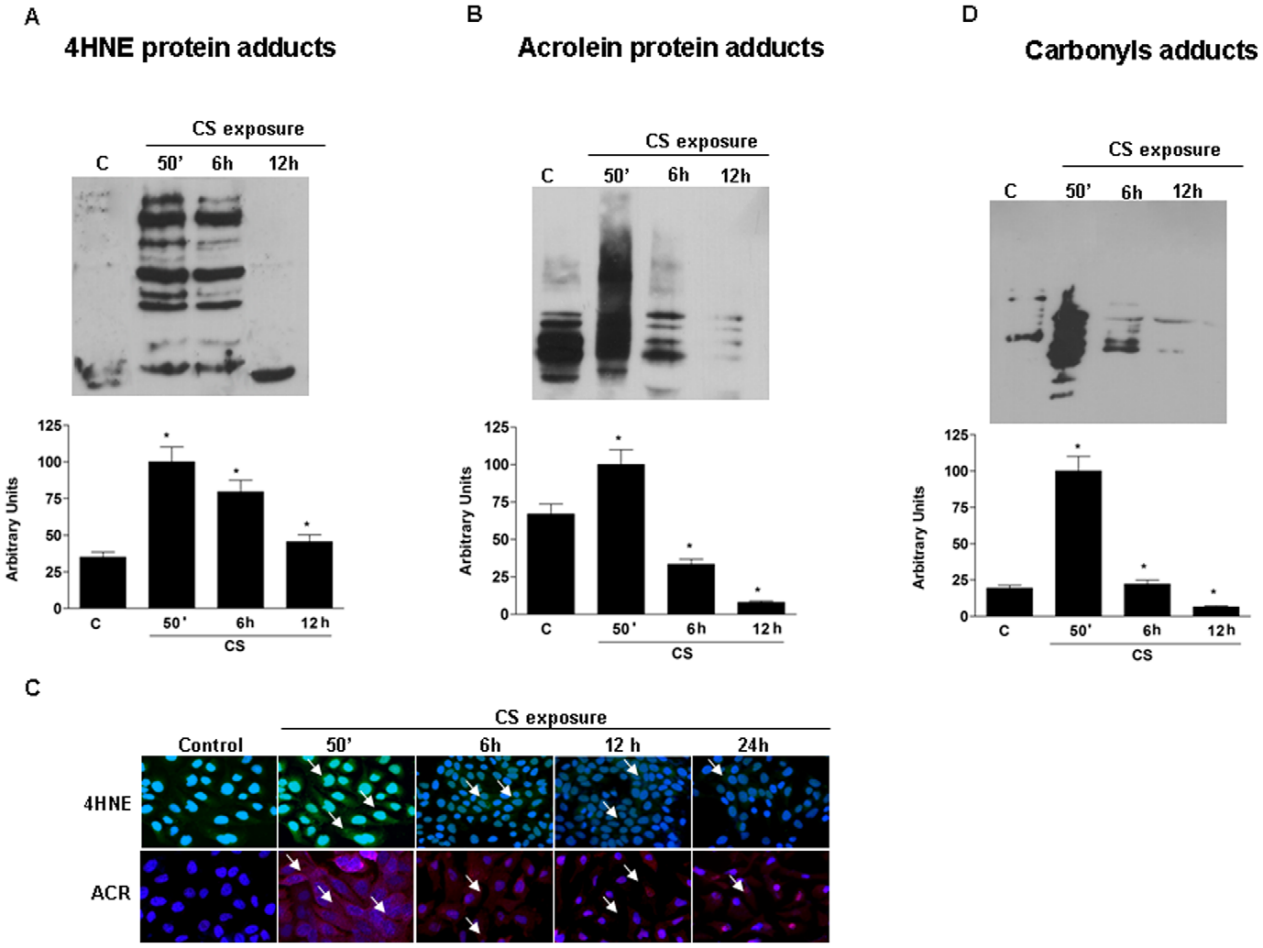
Exposure to CS increased HNE (A) and ACR protein adducts (B) in HaCaT cells measured by Western blot and this is confirmed also by ICC staining for HNE and ACR (C) (see arrows). CS increased carbonyl groups expression (D) in HaCaT cells. Cells were exposed to CS for 50 min and cells were harvested at different time points (0–12 hrs). Western blot shown in the top is representative of five experiments. Quantification of the SR-B1 bands is shown as ratio of SRB1/β-actin (bottom panel). Data are expressed as arbitrary units (averages of five different experiments, *p<0.05; **p<0.01). β-actin was used as loading control.

Many of the toxic effect of CS can be linked to the presence of aldehydes, therefore we have evaluated the levels of acrolein (ACR) and HNE adducts in keratinocytes exposed to CS. As shown in Fig. 4A after CS exposure there was a significant increase of HNE protein adducts levels. This increase was evident immediately after the exposure to CS exposure (3 fold) and although this declined over time, it was still significant at 12 hrs (almost 2 fold increase over control). Parallel results were observed also for ACR protein adducts (Fig. 4B) even if the results were less dramatic with a gradually decreased to the control level at 50 minutes of CS exposure. These results were also confirmed by immunocytochemistry (ICC) as shown in Fig. 4C. Michael addition of α,β-unsaturated aldehydes to cys, his or lys residues adds an aldehyde function to proteins but exposure to CS could also lead either to direct oxidative modification of proteins including formation of carbonyls groups. Therefore, we also examined the levels of protein carbonyls group in keratinocytes exposed to CS by Oxyblot analysis. As shown in Fig. 4D, CS exposure induced a significant increase in carbonyls levels (4 fold) compared to the control.

### Effect of HNE and ACR on SRB1 levels

**Figure 5:**
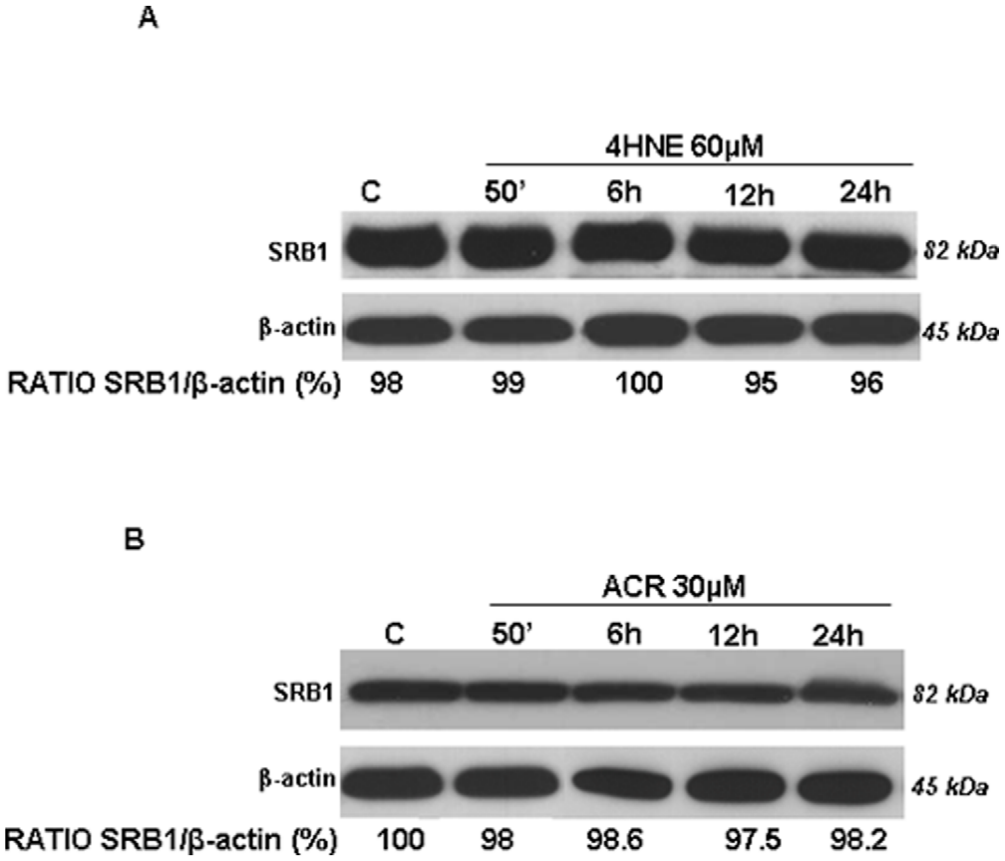
HNE (A) or ACR (B) treatment did not affect SR-B1 levels in HaCaT cells. Cells were exposed to the different treatments for 50 min and cells were harvested at different time points (0–24 hrs). Numbers below the blot represent the ratio of SRB1/β-actin quantification.

To examine whether exogenous HNE or ACR affected SR-B1 protein levels, HaCaT cells were treated with different concentration (10 to 100 uM) of either HNE or ACR. As showed in Fig. 5A, 60 µM of HNE treatment did not affect SR-B1 expression (the same results were seen also with concentrations from 20 to 100 µM – data not shown). Parallel results were observed when the cells were treated with ACR. As shown in Fig. 5B, no changes in SR-B1 levels were detected after treatment with 30 µM of ACR (same results were obtained with doses from 10 to 70 µM – data not shown). This unexpected result suggested that the carbonyl formation and aldehyde adducts detected after CS exposure is a consequence of a cellular response to CS.

### CS exposure induced HNE/SR-B1 adducts

**Figure 6:**
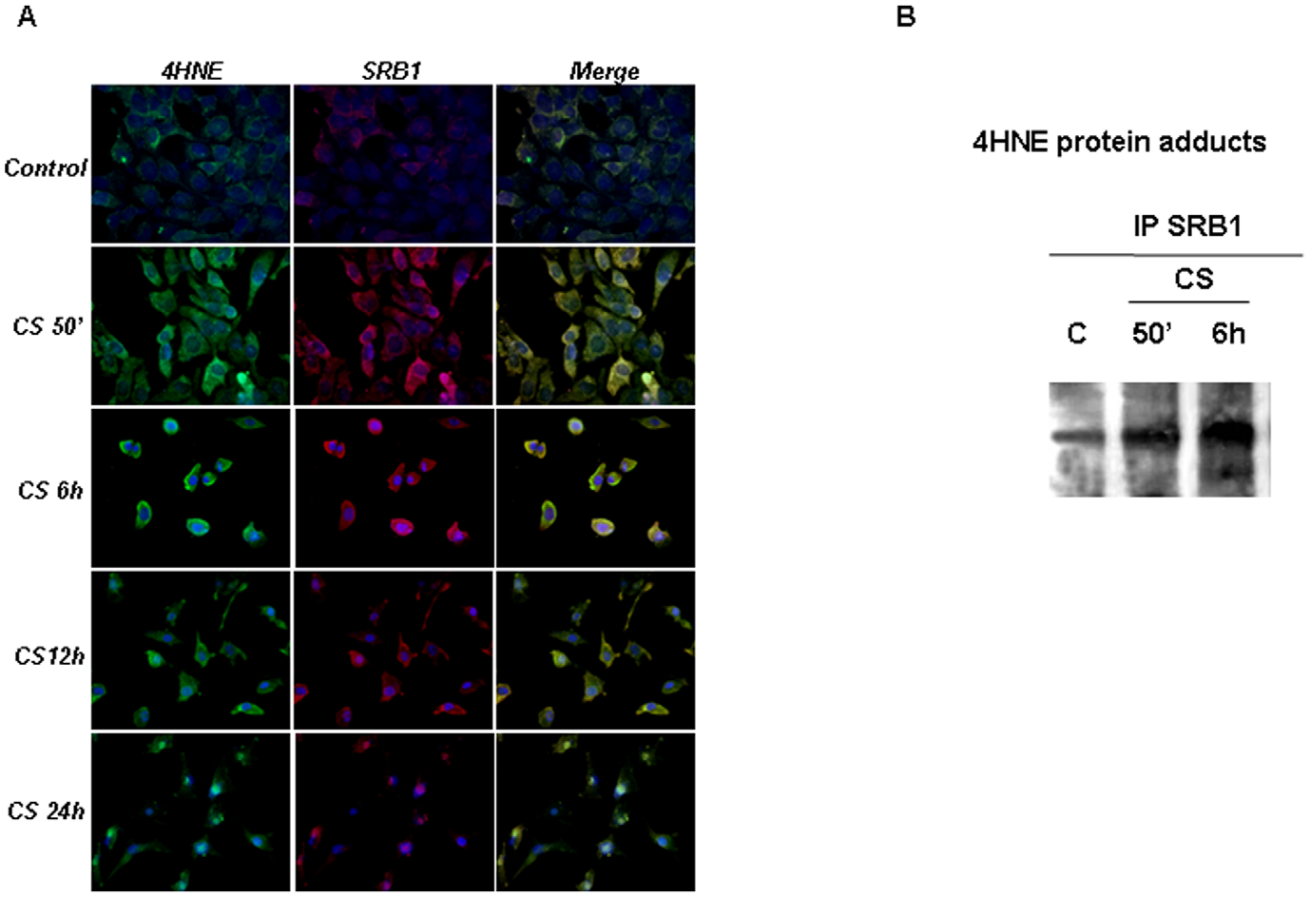
CS induces the increase of HNE/SRB1 adducts. Immunocytochemistry of HaCaT cells showing localization of HNE-adducts (left column, green color), SR-B1 (central column, red color) and HNE/SR-B1 adducts (right column, yellow color) before, and several time points after, CS exposure (A). Images are merged in the right panel and the yellow color indicates overlap of the staining. These data were confirmed by immunoprecipitation for SR-B1 (B). HaCaT cells were exposed to CS and cell lysates were immunoprecipitated using anti SR-B1. Immunoprecipitated proteins were separated by SDS-PAGE, and then transferred to a nitrocellulose membrane and immunoblotted with anti-HNE. Western blot shown is representative of five independent experiments.

We evaluated whether the formation of HNE protein adducts in cells exposed to CS included modification of SR-B1. As shown in Figure 6A, after CS exposure, the levels of HNE increased dramatically (left column) with a concomitant decrease of SR-B1 (Figure 5 central column). The co-localization (yellow) appreciable in the right column showed the presence of HNE adducts on SR-B1. These data suggested that CS induced a covalent modification of SR-B1 via Michael addition of HNE. To confirm this result, we employed antibodies against HNE–protein conjugates in combination with SR-B1 antibody in reciprocal immunoprecipitation–Western blot analysis. As shown in Figure 6B, the IP experiments showed the interaction between SR-B1 and HNE. The IP results were less dramatic than the immunocytochemistry most likely because SR-B1 is only one of many proteins modified by HNE as shown in Fig. 4A.

### CS exposure induced Ubiquitin/SR-B1

**Figure 7:**
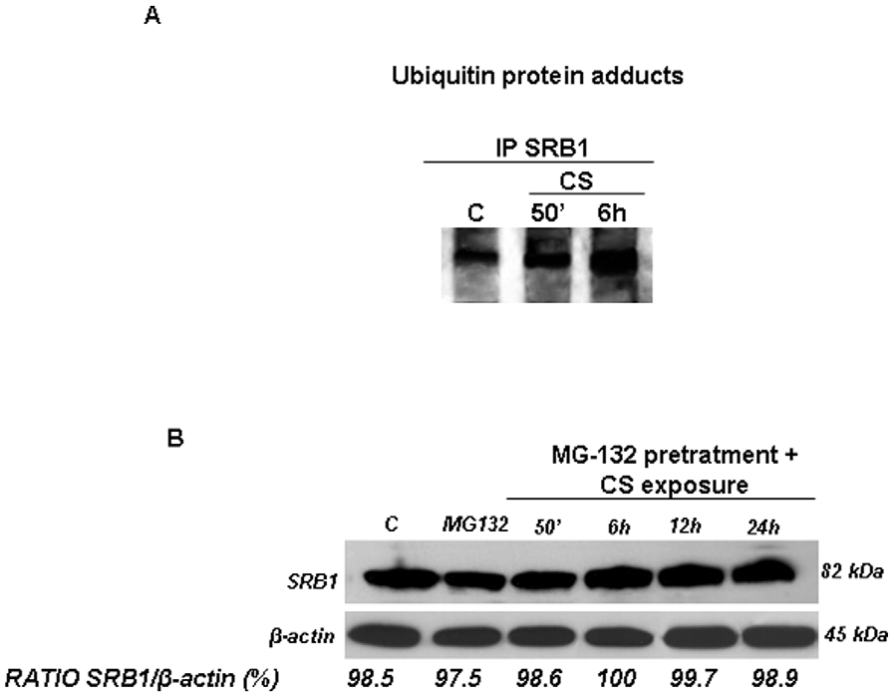
CS induces the increase of Ubiquitin/SR-B1 adducts. HaCaT cells were exposed to CS and cell lysates were immunoprecipitated using anti SR-B1. Immunoprecipitated proteins were separated by SDS-PAGE, and then transferred to a nitrocellulose membrane and immunoblotted with anti-Ubiquitin (A). Pretratment (2 h) with MG-132 (proteosome inhibitor) did not affect SR-B1 levels. Cells were exposed to CS for 50 min and harvested at different time points (0–24 hrs). Western blot shown in the top is representative of five independent experiments. Quantification of the SR-B1 bands is shown as ratio of SR-B1/β-actin (bottom panel). Data are expressed as arbitrary units (averages of five different experiments). β-actin was used as loading control.

We next evaluated whether CS also induced an increase of SR-B1 ubiquitination in keratinocytes [25]. Immunoprecipitation for SR-B1 ubiquitination revealed that CS exposure increased ubiquitinated SR-B1 in HaCaT cells after 50 min of CS exposure (Fig. 7A). Re-probing of the membrane with anti SR-B1 antibodies indicated that equivalent amounts of SR-B1 were immunoprecipitated from each sample (data not shown). These data were confirmed using an inhibitor of the proteosome (MG132). CS exposed cells in the presence of MG132 did not show changes in SR-B1 levels (Fig. 7B).

### H_2_O_2_ decreased SR-B1 protein levels

**Figure 8:**
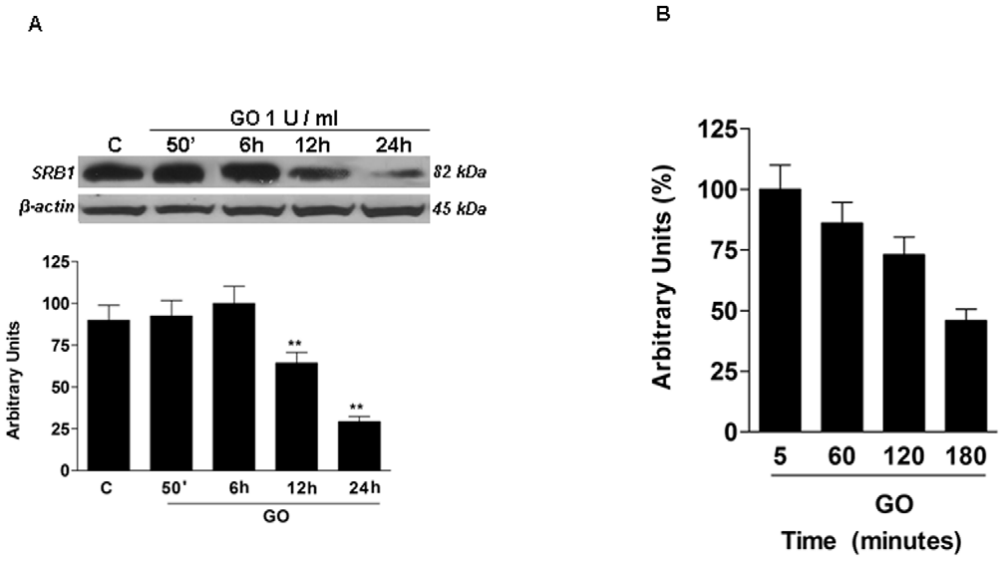
GO treatment decreased SR-B1 levels. Cells were treated with GO for 50 min and then harvested at different time points (0–24 hrs). A) Representative Western blot of five independent experiments is shown in the top panel. Quantification of the SRB1 bands, average of the five independent experiments, is shown in the bottom panel. Data are expressed in arbitrary units (**p<0.01). β-actin was used as loading control. B) Concentration of H_2_O_2_ level in cell treated with GO. Data are presented as average of triplicate measurements from each sample and expressed as arbitrary units.

It has been shown that H_2_O_2_ is among the most reactive oxidants present in the gas phase of CS [30]. The results above suggested that aldehydes produced in the CS exposed cells rather in the CS itself were involved in SR-B1 modification and loss. Therefore, the possibility that H_2_O_2_ in CS or produced by cells in response to CS exposure mediated SR-B1 modification and loss was considered. As shown in Fig. 8A, H_2_O_2_ generated by glucose oxidase (GO) induces a significant decrease in SR-B1 levels. The effect was significant after 12 hrs and was even more dramatic after 24 hr. As these results were similar to that shown by CS exposure in Fig. 2, we hypothesized that the effect of CS on SR-B1 levels was mainly driven by H_2_O_2_. As shown in Fig. 8B the levels of H_2_O_2_ generated by GO were around 100 µM and decreased by 50% after 3 hrs.

### CS exposure increased cellular H_2_O_2_ production

**Figure 9:**
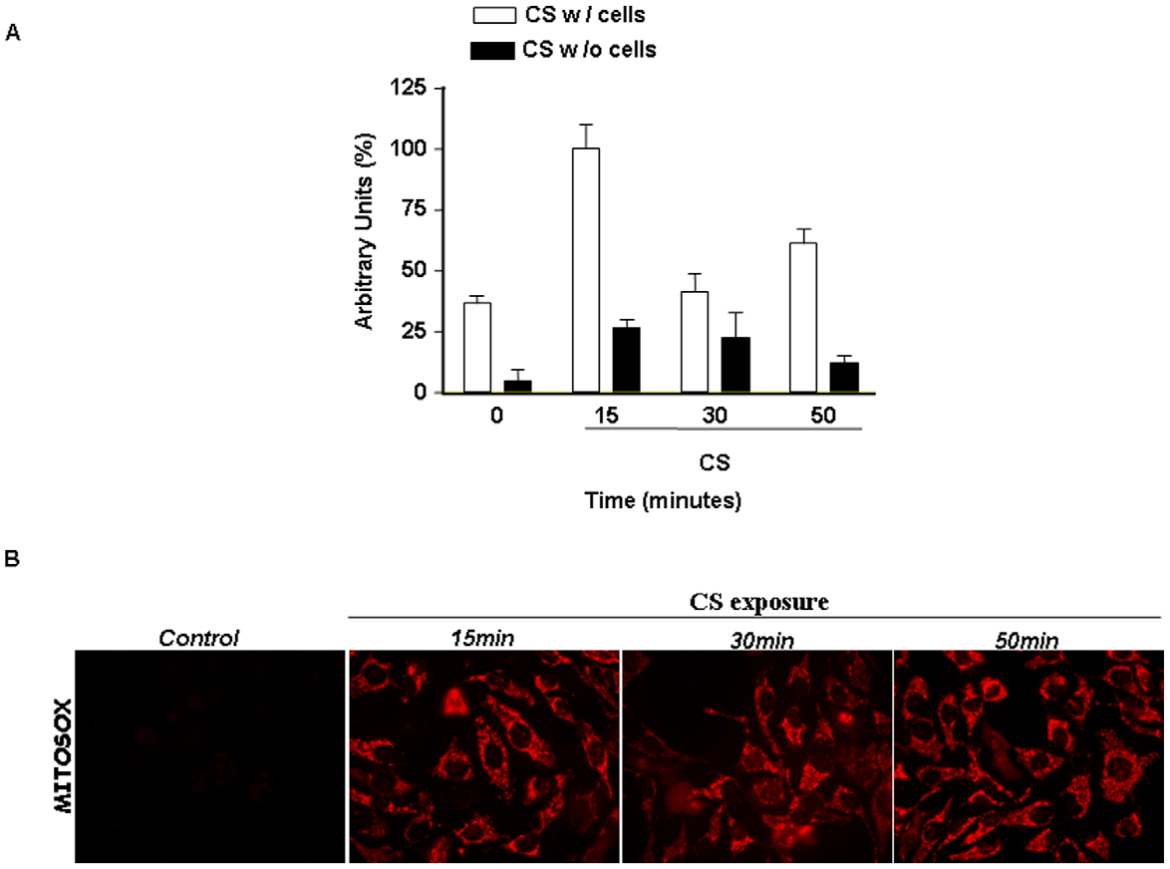
CS exposure increased H2O2 levels and mitochondrial superoxide production. Cells were exposed to CS for 15, 30 or 50 min. (A) Concentration of H_2_O_2_ in the media with (close bars) or without cells (open bars). Data are presented as average of triplicate measurements from each sample and expressed as arbitrary units. (B) Mitochondrial ROS production was evaluated by Mitosox fluorescence. Cells were loaded with Mitosox before and after CS exposure and subjected to live cell imaging.

To further test the hypothesis that H_2_O_2_ was responsible for the effect of CS exposure, we analysed the production of H_2_O_2_ during CS exposure. After 15 min of treatment, there was a significant increase (2 fold) of H_2_O_2_ in the cells exposed to CS and H_2_O_2_ then declined to the steady level after 30 min (Fig. 9). Of note is that when the media without the cells was exposed to CS the level of H_2_O_2_ detected was lower then when the cells were present in the media. This suggests that the cells contributed to the generation of H_2_O_2_.

### CS exposure increased mitochondrial ROS production

Mitochondrial ROS production was evaluated in HaCaT cells after CS exposure. The fluorescent dye Mitosox Red, which has been suggested to detect mainly mitochondrial superoxide [31], was used for these experiments. As shown in Figure 9B, cells exposed to CS showed an increased of fluorescence characteristic of mitochondrial staining, that is maintained throughout the smoke exposure (15–50 min), whereas, control cells exposed to air did not show this effect.

### CS exposure induced translocation of the NADPH oxidase (NOX) components, p67^phox^ and p47^phox^

**Figure 10:**
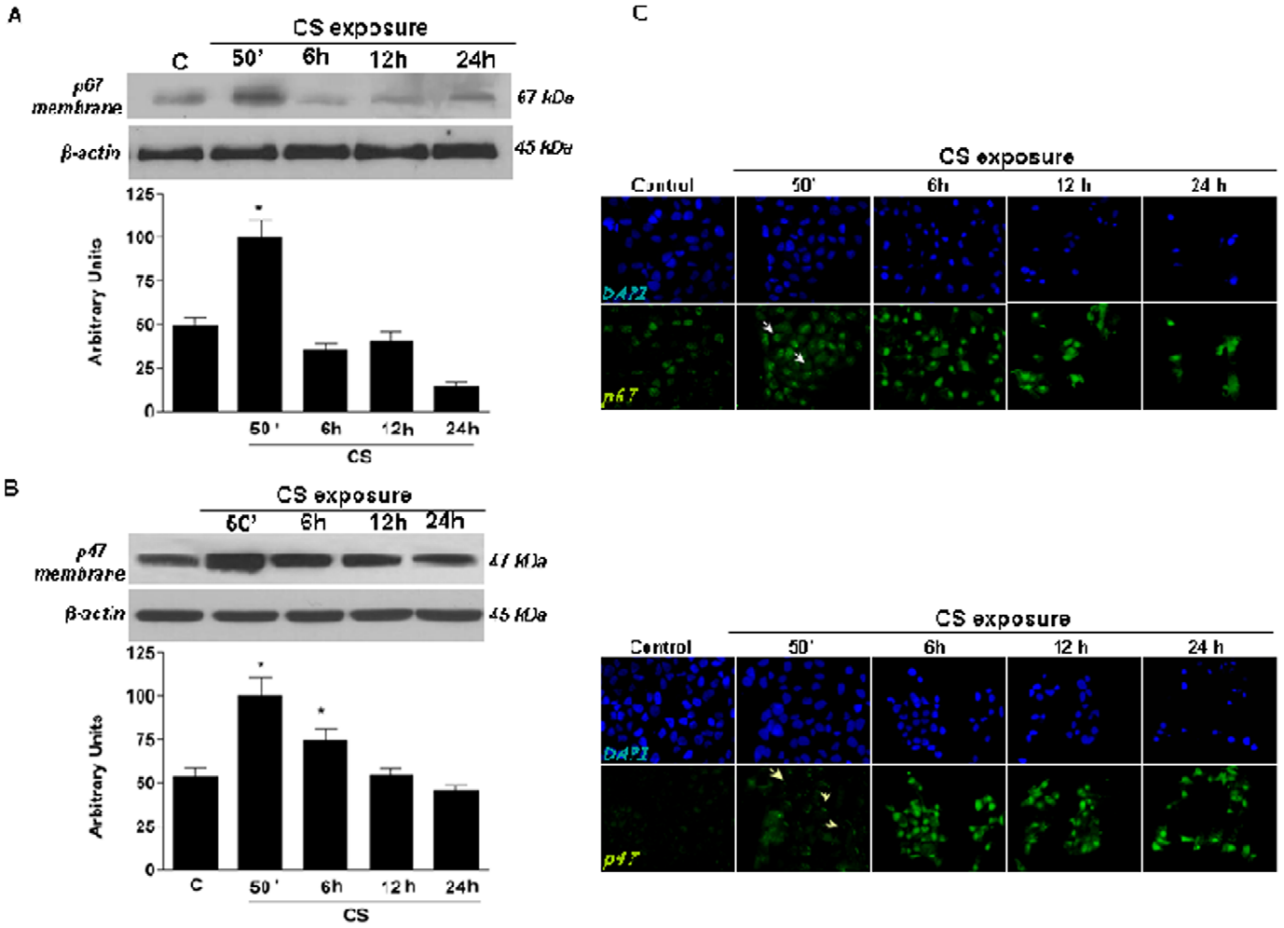
Exposure to CS increased NADPH oxidase levels in HaCaT cells. Cells were exposed to CS for 50 min and cells were harvested at different time points (0–24 hrs). The activation of NADPH oxidase was determined by the translocation in membrane of p67*^phox^* (A) and p47*^phox^* (B). The Western blot shown in the top is representative of five experiments. Quantification of the SR-B1 bands is shown in the bottom panel. Data are expressed as arbitrary units (averages of five different experiments, *p<0.05). β-actin was used as loading control. These data were confirmed by ICC for p6*^phox^* and p4*^phox^* (C).

One possible cellular source of H_2_O_2_ is NADPH oxidase (NOX) [32], therefore we investigated whether CS exposure induced activation of this enzyme. As shown in Figure 10, the membrane levels of the cytoplasmic subunits p67^phox^ (panel A) and p47^phox^ (panel B) were significantly increased after CS exposure, demonstrating the activation of NOX. These effects were also confirmed by immunocytochemistry (ICC) as shown in Fig. 10C.

### Catalase and DPI pretreatment prevented decreased SR-B1 levels induced by CS exposure

**Figure 11:**
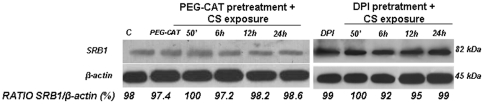
The decreased levels of SR-B1 after CS exposure was reversed by catalase (CAT) (left panel) or Diphenyleneiodonium Chloride (DPI) (right panel). Cells pretreated with CAT or DPI were exposed to CS for 50 min and harvested at different time points (0–24 hrs). Western blot shown is a representative of five independent experiments. Quantification of the SR-B1 bands is expressed under the blot as ratio of SR-B1/β-actin (arbitrary units).

To confirm that the decreased level of SR-B1 after CS exposure was mainly driven by the production of H_2_O_2_, HaCaT cells were pretreated with PEG-catalase (PEG-CAT) and then exposed to CS. As shown in Fig. 11, CAT pretreatment prevented the decrease of SR-B1 levels caused by CS exposure. To support the involvement of NOX, HaCaT cells were pretreated with DPI (a general inhibitor of flavoproteins including NOX) and exposed to CS. As shown in Fig. 11 (right panel), DPI pretreatment largely prevented the decrease of SR-B1 levels induced by CS exposure.

## Discussion

The results presented in this study suggested a possible mechanism through which CS induced modification, translocation and degradation of SR-B1 in keratinocytes.

Many of the recent studies on SR-B1 have been focused on its interaction with HDL and on its role in mediating the selective uptake of HDL cholesterol esters especially in steroidogenic tissues and liver [1], [2], [33]–[35]. The physiological role of SR-B1 has been tested *in vivo* and *in vitro* by genetic manipulation and has been shown that mice lacking of SR-B1 have an impaired hepatic-selective HDL cholesterol uptake, suggesting its role in hepatic cholesterol transfer. In the last few years it has been shown that SR-B1 plays a wider role in cell cholesterol levels and can affect the levels of free cholesterol in the plasma membrane and therefore influences the cell membrane structure [35], [36]. In addition, other functions of this receptor have been shown. Although not directly connected to its ability to recognize HDL, SR-B1 has been shown to regulate calcium permeability in lymphocytes [35], [37], and be involved in bacteria recognition [35], [38] and vitamin E tissue uptake [35], [39], [24]. SR-B1 has also been shown to be expressed in several other tissues rather than liver, including lung, ovary, testis, brain, spleen, kidney [39] and, recently, even skin [40]. The role of SR-B1 in cutaneous tissue could be related to its ability to regulate cholesterol trafficking as suggested by the work Tsuruoka et al. [40] in which SR-B1 levels decreased as the keratinocytes differentiated but increased after insults, such as tape stripping, as the epidermis required more lipids to restore the permeability barrier. It is quite possible that many other functions of SR-B1 in skin may be discovered. Indeed, it was surprising to find out that SR-B1 was heavily expressed in the epidermis which is the less vascularized part of cutaneous tissue and therefore less exposed to HDL particles. It has been shown that among the insults to which the skin is exposed, CS is one of the most toxic [41] and in addition, passive smoke (sidestream smoke) is even more toxic than the mainstream smoke, based on its chemical composition [42]. Environmental CS contains not only a large amount of oxygen (reactive oxygen species: ROS) and nitrogen (reactive nitrogen species: RNS) radical forming substances [43], but also very reactive aldehydes such as ACR which is known to disturb biological systems by reacting with a variety of constitute molecules [19]. In our work we have shown a clear increase of carbonyls and of both, ACR and HNE protein adducts after CS exposure. This is the consequence of the high reactivity of the α,β-unsaturated aldehydes to form covalent bounds with amino acids residues such as lysine, histidine and cysteine presents in the proteins [21]. After CS exposure there was an evident HNE/SR-B1 adducts formation, showing that SR-B1 is one of the protein target of α,β -unsaturated aldehydes. The presence of ACR and HNE protein adducts has been connected with both skin aging and inflammation as shown in the work of Tanaka et al. [44] where immunohistochemical analysis for HNE and ACR in skin were associated with actinic elastosis.

Since treatment with the aldehydes HNE or ACR at different concentrations did not affect the levels of SR-B1 we have addressed the possibility that H_2_O_2_ could be the component present in CS able to induce the modulation of SR-B1 as suggested also in other studies [18]. In fact, CS contains both acrolein and H_2_O_2_; however in our results we have noticed that while exogenous ACR did not affect SR-B1 levels, H_2_O_2_ was able to reproduce the same pattern observed after CS exposure with a dramatic decrease of SR-B1 expression. This effect was inhibited by catalase pretreatment therefore H_2_O_2_ was most likely the mediator able to modify SR-B1 levels.

We have noticed that the concentration of H_2_O_2_ in CS (when only the medium was exposed to CS) was lower than that in the presence of cells, causing us to conclude that part of the H_2_O_2_ present in the system derived from the cells (endogenous) and not only from the gas phase present in CS (exogenous). This was confirmed by the detection of NOX activation, which is involved in H_2_O_2_ production via the generation of superoxide (O_2_^−^). In addition, NOX has been shown to be inducible by environmental stressors [45]. Although it has been shown that the general flavoproteins inhibitor DPI can induce cell death [46], [47], it can be cautiously used as NOX inhibitor. In our study, cells pre-treated with DPI showed an attenuated SR-B1 loss, suggesting that CS-induced cellular H_2_O_2_ production played a major role in SR-B1 loss. NADPH oxidase uses NADPH to produce superoxide anion (O_2_^−^) and consists of plasma membrane-bound subunits (gp91^phox^/Nox2 and p22^phox^) and cytosolic subunits (p40^phox^, p47^phox^, p67^phox^, Rac1) that assemble at the membrane to produce the active enzyme after a stimulus [48]. Our results demonstrate that after CS exposure, both p67*^phox^* and p47*^phox^* were increased in the membrane-bound fraction, which is an indicator of NADPH-oxidase activation [49]. These data are in agreement with the study by Chamulitrat et al [50], which showed that HaCaT express NOX subunits that have the ability to generate O_2_^−^ when the cell membrane was isolated. Although controversial, the use of mitosox can be use to measure the presence of mitochondrial O_2_^−^. Our data showed that after CS there was a clear increased of the red dye suggesting that CS induces also mitochondrial oxidative stress which is in agreement with previous studies like the one from van der Toorn M et al. [51] has shown that lipophilic components present in cigarette smoke extract such as polycyclic aromatic hydrocarbons, phenols and aldehydes, which does not contain ROS, are able to pass through the membranes and subsequently disturb mitochondria and this could fit with our data.

Of note is that when HaCaT cells were compared with neutrophils (PMNs), keratinocytes produced 20 time less O_2_^−^ than the neutrophils but the K_m_ of keratinocytes membranes (NOX) was almost a factor of 2 higher than PMNs supporting the idea that keratinocyte NOX generates a constitutively constant level of O_2_^−^ [52]. Activation of NOX in keratinocytes has been shown to be involved in both migration and proliferation, therefore playing a critical role in skin physiology. Interestingly, Nam et al. [53] showed that enhanced cell migration was dependent on H_2_O_2_ generation mediated by NADPH oxidase but that cell migration was not enhanced by treating the cells with H_2_O_2_ directly. This supports the idea that more than the concentration of H_2_O_2_ is important the source and the rate of production. Indeed, signaling by H_2_O_2_ is very much localized phenomenon in which the location of the source and target and the rate of production are critical [54]. This is also applicable to the aldehydes. In fact, the use of HNE or ACR did not affect SR-B1 levels while exposure to CS leads to the increase of HNE and ACR (in part endogenously) that can then form protein adducts with SR-B1. It has been recently shown that HNE production is able to directly activate NOX [55], therefore it is possible that the peroxidation products induced by CS are the responsible for NOX activation also in our system.

The role of H_2_O_2_ generated by CS in modification of receptors has been shown in other cells [56], [18]. Here, we demonstrated that CS affected SR-B1 levels and localization in keratinocytes via the activation of NOX with the production of H_2_O_2_, and the subsequent formation of SR-B1/aldehyde adducts that led to the ubiquitination and degradation of the receptor. Similar results where also shown by our lab in epithelial lung cells although the mechanism that contributes to the loss of SR-B1 was not clear. Now we have shown that the decline of SR-B1 was due to the formation of SR-B1 protein adducts and by increased ubiquitination, which led to SR-B1 degradation by the proteosome. Indeed, the use of the proteosome inhibitor MG132 reversed the effect.

The presence of RNS in CS has been well documented [57] and it is possible that others posttranslational modifications (Nitrotyrosine) play a role in SR-B1 levels. The activation by CS of NOX with the release of O_2_^−^ and iNOS [58] with the production of NO could lead to the formation of peroxynitrite (ONOO^−^) a very reactive molecule that can oxidized sulphhydrils about 10^3^ times faster than H_2_O_2_ [59] and might contribute to SR-B1 modifications.

**Figure 12:**
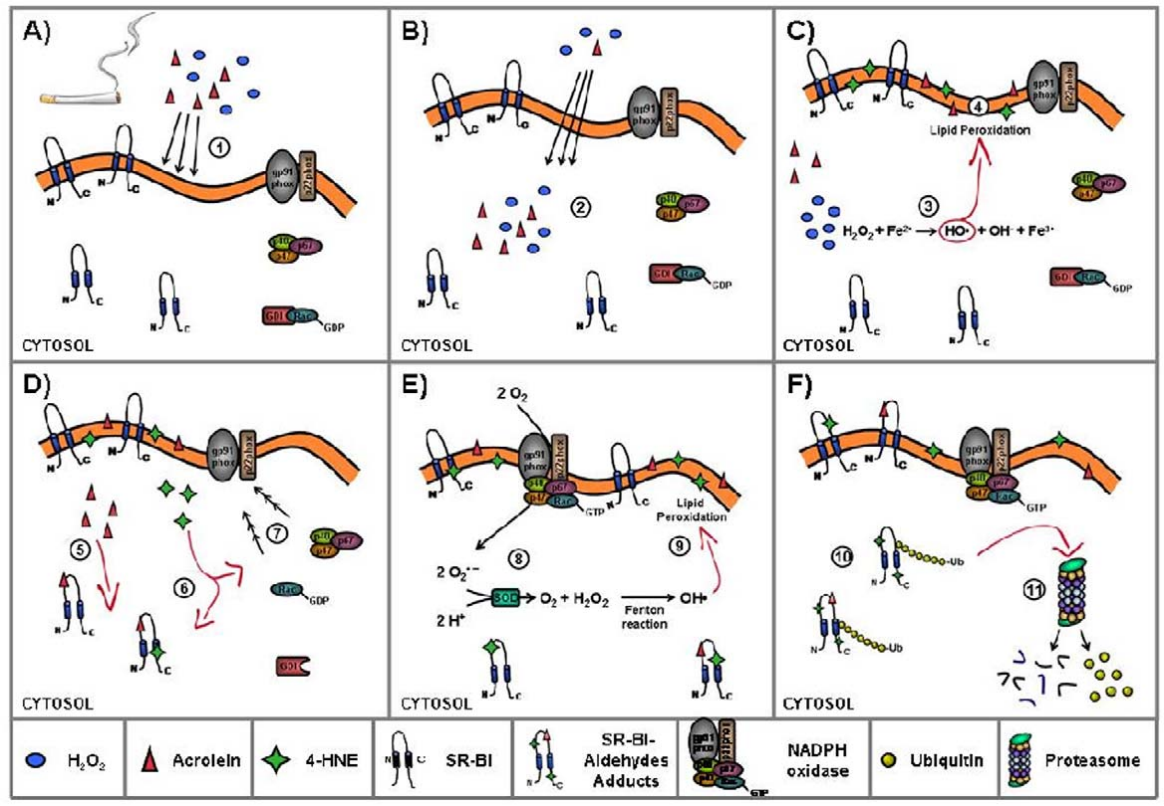
Possible mechanism involved in the degradation of SR-B1. Among the components present in CS there are acrolein and H_2_O_2_ that beside to react with the membrane lipids (1) are able to cross the cell membrane (2), once H_2_O_2_ is inside the cells, there will be the formation of OH. (Fenton reaction) (3) that will react with the cytosolic membrane lipids and the formation of lipid peroxidation products such as ACR and HNE (4). ACR and HNE can from SR-B1 adducts (5 and 6) and HNE can also activate NOX by inducing the translocation of the cytoplasmic submit to the membrane (7). Activation of NOX lead to the increased production of O_2_^−^ that can be dismutated (SOD) in H_2_O_2_ (8) that via Fenton reaction will further increase the level of peroxidation (9). The formation of HNE-SR-B1 adducts is recognized by the ubiquitination apparatus of the cell (10) that will ubiquitinate the protein that subsequent will be dregraded by the proteosome (11).

How does H_2_O_2_ cause the modification and loss of SR-B1? Consistent with the data here, H_2_O_2_ appears to have caused the production of ACR and HNE from cellular components. HNE and ACR can be produced from lipid peroxidation while ACR can come from the oxidation of carbohydrates as well. Regardless, the formation of ACR and HNE adducts of SR-B1 was associated with translocation, ubiquitination, and degradation of SR-B1. The observation that cellular production of these α,β-unsaturated aldehydes resulted in SR-B1 loss while exogenous addition did not, suggests that the location of the aldehyde production was important. It is possible that recognition of the adducted protein by the ubiquitination apparatus of the cell required modification of the protein on the cytosolic domains of the receptor while modification on the external side would not have been recognized. Thus, production of the aldehydes from lipids on the cytosolic side of the plasma membrane local to the SR-B1 already in the membrane or from internal cellular membranes, particularly the endoplasmic reticulum where SR-B1 was also present prior to CS exposure was able to modify SR-B1 in a manner that caused its modification and subsequent ubiquitination and degradation (Figure 12).

In conclusion, considering the noxious effect of CS on cutaneous tissues, our data bring new insights on the possible mechanism by which CS exposure leads to the loss of SR-B1 receptor, which plays a prominent role in the delivery of lipids from the extraepidermal tissues to epidermis and thereby contributes to the cutaneous barrier via the formation of lamellar bodies. Although this receptor has been studied mainly for its function to recognized HDL particles, several other functions have been described and hypothesized therefore it is not surprising that it would also have a prominent role in regulating skin physiology.
