# Correction: Cigarette Smoke Affects Keratinocytes SRB1 Expression and Localization via H_2_O_2_ Production and HNE Protein Adducts Formation

**DOI:** 10.1371/journal.pone.0228663

**Published:** 2020-01-30

**Authors:** Claudia Sticozzi, Giuseppe Belmonte, Alessandra Pecorelli, Beatrice Arezzini, Concetta Gardi, Emanuela Maioli, Clelia Miracco, Marzia Toscano, Henry Jay Forman, Giuseppe Valacchi

After publication of this article [1], concerns were raised regarding western blot panels in Figs 2A, 5A and 5B. Specifically, it was noted that there appears to be a vertical discontinuity between lanes 1 and 2 of the SRB1 panel in Figure 5A, and that the β-actin panels in Figs 5B and 2A are highly similar, although with different aspect ratios.

**Fig 2:**
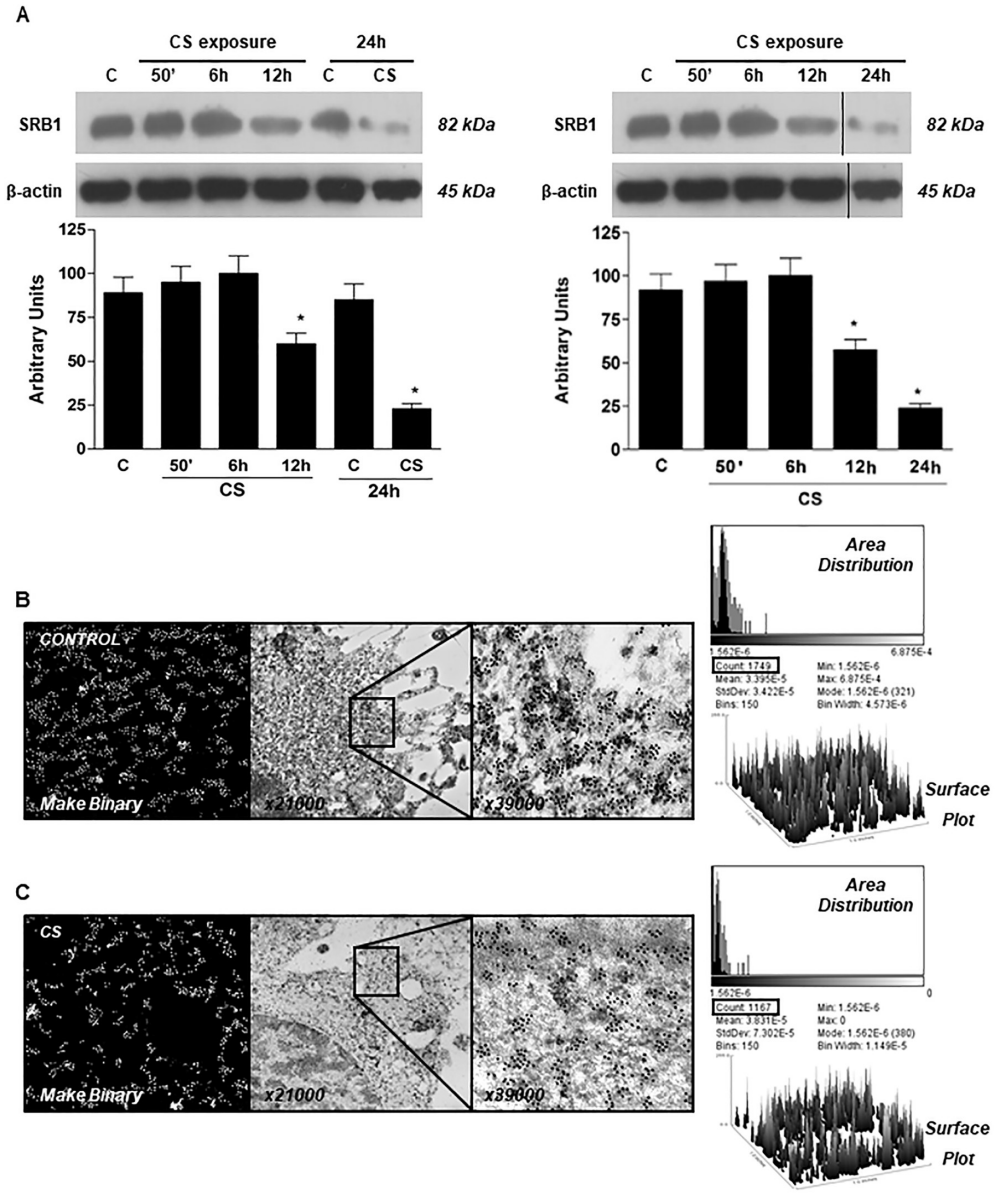
Exposure to CS decreased SR-B1 protein levels in HaCaT cells. Cells were exposed to CS for 50 min and cells were harvested at different time points (0–24 hrs). The Western blot shown in the top is representative of five experiments. Quantification of the SR-B1 bands is shown in the bottom panel. Data are expressed in arbitrary units (averages of five different experiments, *p<0.05). β-actin was used as loading control. Immunogold for SR-B1 confirm the decreased protein levels after CS exposure (B). IHC for SR-B1 is shown in the C panel (arrows).

The authors note that the original blot underlying the SRB1 panel of Figure 5A is no longer available, and they are unable to clarify the seeming discontinuity between lanes 1 and 2 of this figure. Confirmatory data from replication experiments are provided in S1 and S2 Files of this notice.

Regarding similarities between the β-actin panels in Figs 2A and 5B, the authors clarified that they erred in preparing the manuscript such that the β-actin blot from the experiment shown in Figure 5B was included in Fig 2A in error. The authors also clarified that lane 5 of the original SRB1 blot for Fig 2A (a 24-hour control lane) was removed in preparing Fig 2A such that lanes 1–4 and 6 were spliced together in the published figure. The authors provide here an updated version of Fig 2A, including all lanes from the original blots and a β-actin blot for the same protein samples as were used in the SRB1 blot. In addition, replication data for the Fig 2A experiment and the underlying raw blot images and quantitative data are included below in S1 and S3 Files. The authors clarified that the quantification data (S1 File and Fig 2A) relied on the correct β-actin data, not on the duplicated blot shown in the original published figure. The underlying data supporting Figure 5B are no longer available; results from replication experiments are presented in S1 and S2 Files.

The original raw data underlying other figures in this article [1] are no longer available.

The updated version of Fig 2A includes the correct β-actin data, with or without the Control at 24hr. Vertical black lines show where a lane was removed in preparing the version without the Control 24hr data. See S1 File for replication data and S2 File for raw blot images. Quantification was not modified since the quantitative results reported in the original article were obtained originally with the correct loading control data.

## Supporting information

S1 File Replication data supporting Figs 2A, 5A and 5B. The Experiment I blots included for Fig 2A are those presented in the original article (SRB1) and the updated version of Fig 2A with this notice. Quantitative data included here for Fig 2A were obtained by reanalyzing the original blot data, including those in the original published figure. Quantitative data for Figure 5 were obtained using replication data shown in S2 File; the original quantitative and raw blot data for Figure 5 are no longer available.
(PPTX)

S2 File Raw blot images from replication experiments for Figure 5A, 5B. In Exp II° data for Figure 5B, the membrane was cut to enable probing with two antibodies and the SRB1 western blot resulted in high molecular weight non-specific signals. The lower band in this SRB1 blot is the correct size for the SRB1 protein.
(PPTX)

S3 File Raw blot images supporting the results in Fig 2A and the replication results for Fig 2A provided in S1 File. Note that the SRB1 blot on the final slide (labelled “5° exp. 12-01-2010”) includes a lower set of bands not present in other replicates of this experiment. The authors were unable to clarify the identity of these bands.
(PPT)
