# Supplementary material for: Correction: Cigarette Smoke Affects Keratinocytes SRB1 Expression and Localization via H2O2 Production and HNE Protein Adducts Formation
Source: PLoS One. 2020 Jan 30;15(1):e0228663. doi: 10.1371/journal.pone.0228663 (PMC6992178; doi:10.1371/journal.pone.0228663)
Supplement: S1 File — The Experiment I blots included for Fig 2A are those presented in the original article (SRB1) and the updated version of Fig 2A with this notice. Quantitative data included here for Fig 2A were obtained by reanalyzing the original blot data, including those in the original published figure. Quantitative data for Figure 5 were obtained using replication data shown in S2 File; the original quantitative and raw blot data for Figure 5 are no longer available. (PPTX) [file pone.0228663.s001.pptx]

## Slide 1
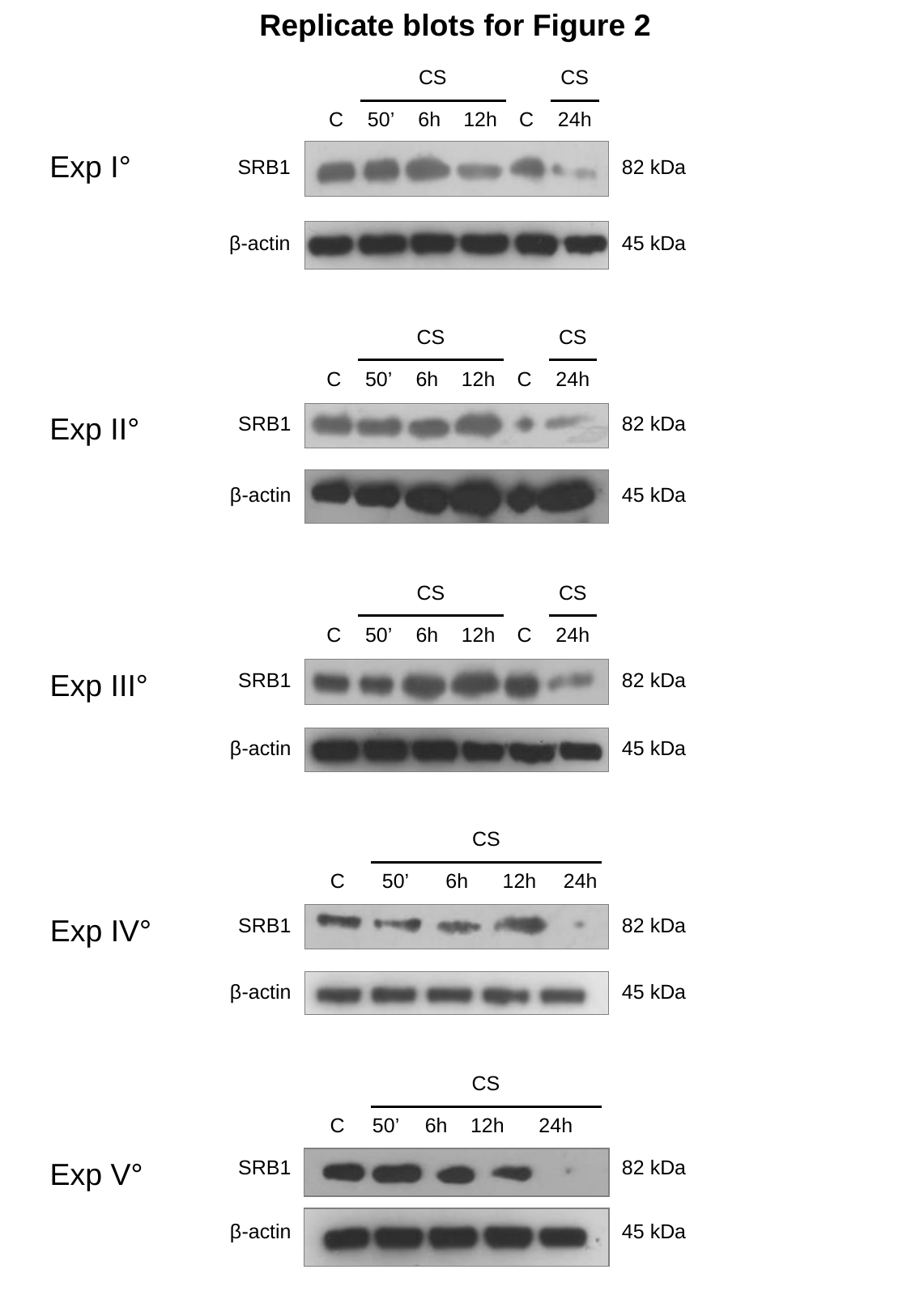

Replicate blots for Figure 2
CS
CS
C
50’
6h
12h
C
24h
Exp I°
SRB1
82 kDa
β-actin
45 kDa
CS
CS
C
50’
6h
12h
C
24h
Exp II°
SRB1
82 kDa
β-actin
45 kDa
CS
CS
C
50’
6h
12h
C
24h
Exp III°
SRB1
82 kDa
β-actin
45 kDa
CS
C
50’
6h
12h
24h
Exp IV°
SRB1
82 kDa
β-actin
45 kDa
CS
C
50’
6h
12h
24h
SRB1
82 kDa
Exp V°
β-actin
45 kDa

## Slide 2
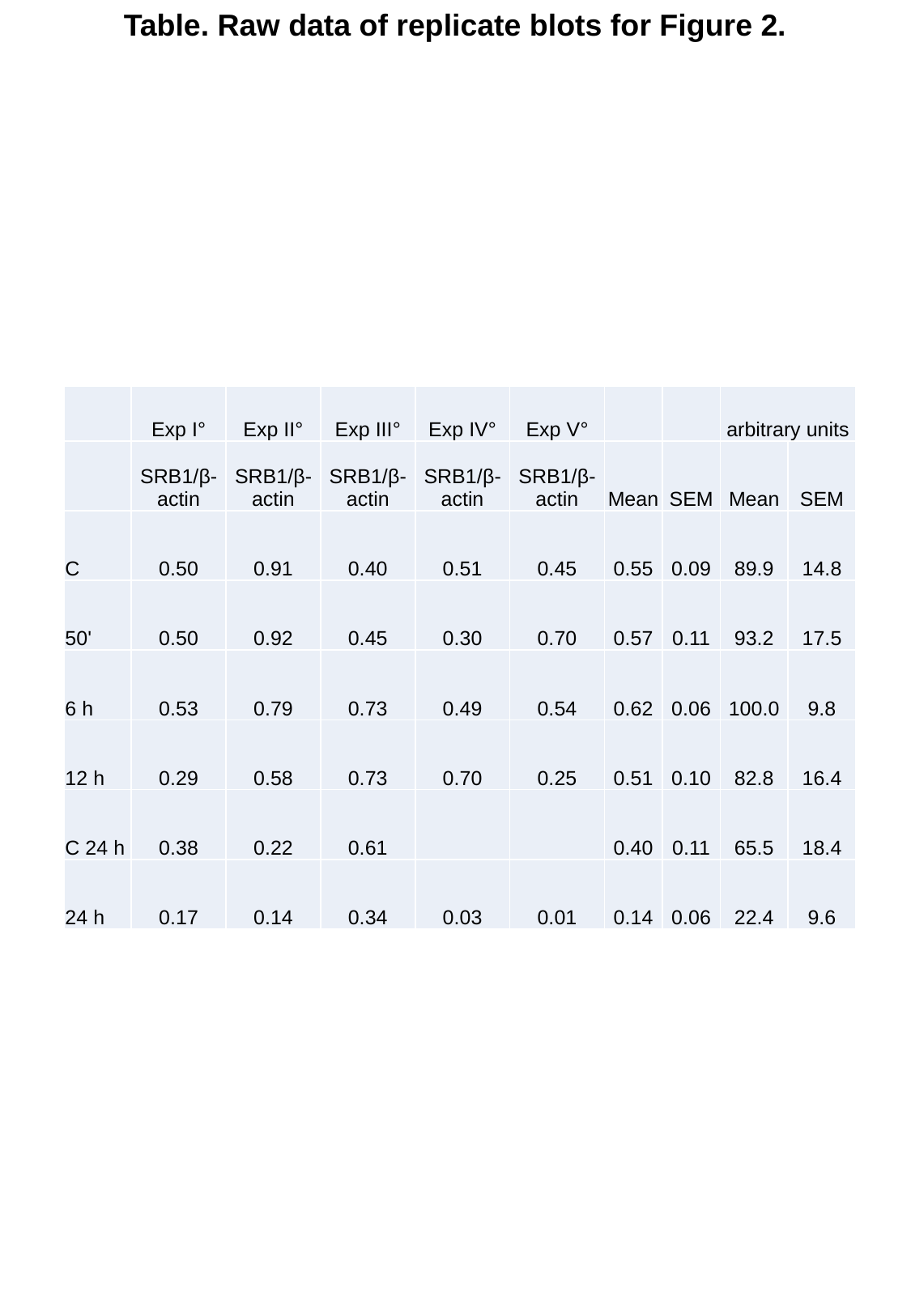

Table. Raw data of replicate blots for Figure 2.
| | Exp I° | Exp II° | Exp III° | Exp IV° | Exp V° | | | arbitrary units | |
| --- | --- | --- | --- | --- | --- | --- | --- | --- | --- |
| | SRB1/β-actin | SRB1/β-actin | SRB1/β-actin | SRB1/β-actin | SRB1/β-actin | Mean | SEM | Mean | SEM |
| C | 0.50 | 0.91 | 0.40 | 0.51 | 0.45 | 0.55 | 0.09 | 89.9 | 14.8 |
| 50' | 0.50 | 0.92 | 0.45 | 0.30 | 0.70 | 0.57 | 0.11 | 93.2 | 17.5 |
| 6 h | 0.53 | 0.79 | 0.73 | 0.49 | 0.54 | 0.62 | 0.06 | 100.0 | 9.8 |
| 12 h | 0.29 | 0.58 | 0.73 | 0.70 | 0.25 | 0.51 | 0.10 | 82.8 | 16.4 |
| C 24 h | 0.38 | 0.22 | 0.61 | | | 0.40 | 0.11 | 65.5 | 18.4 |
| 24 h | 0.17 | 0.14 | 0.34 | 0.03 | 0.01 | 0.14 | 0.06 | 22.4 | 9.6 |

## Slide 3
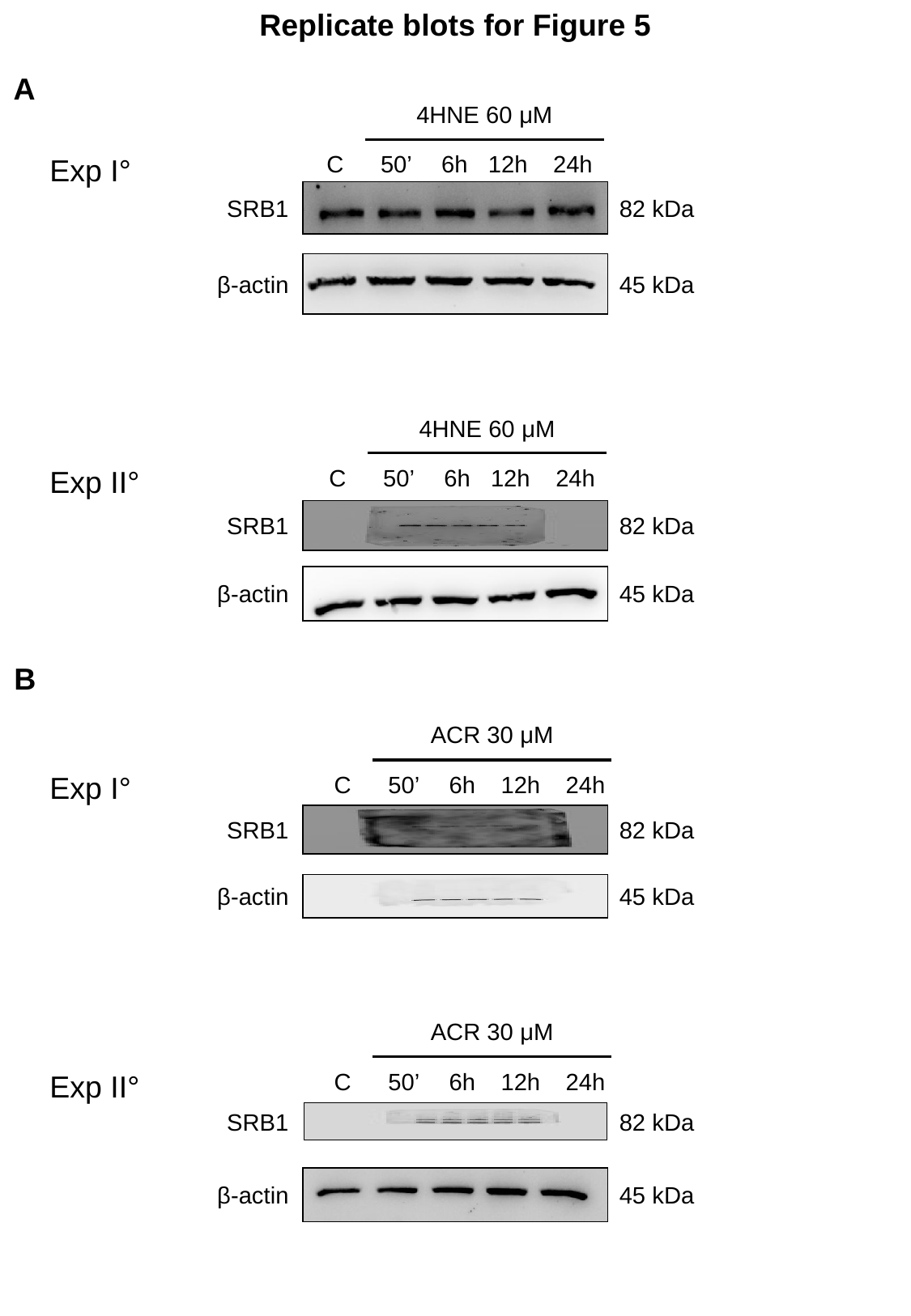

Replicate blots for Figure 5
A
4HNE 60 μM
C
50’
6h
12h
24h
Exp I°
SRB1
82 kDa
β-actin
45 kDa
4HNE 60 μM
C
50’
6h
12h
24h
Exp II°
SRB1
82 kDa
β-actin
45 kDa
B
ACR 30 μM
Exp I°
C
50’
6h
12h
24h
SRB1
82 kDa
β-actin
45 kDa
ACR 30 μM
C
50’
6h
12h
24h
Exp II°
SRB1
82 kDa
β-actin
45 kDa

## Slide 4
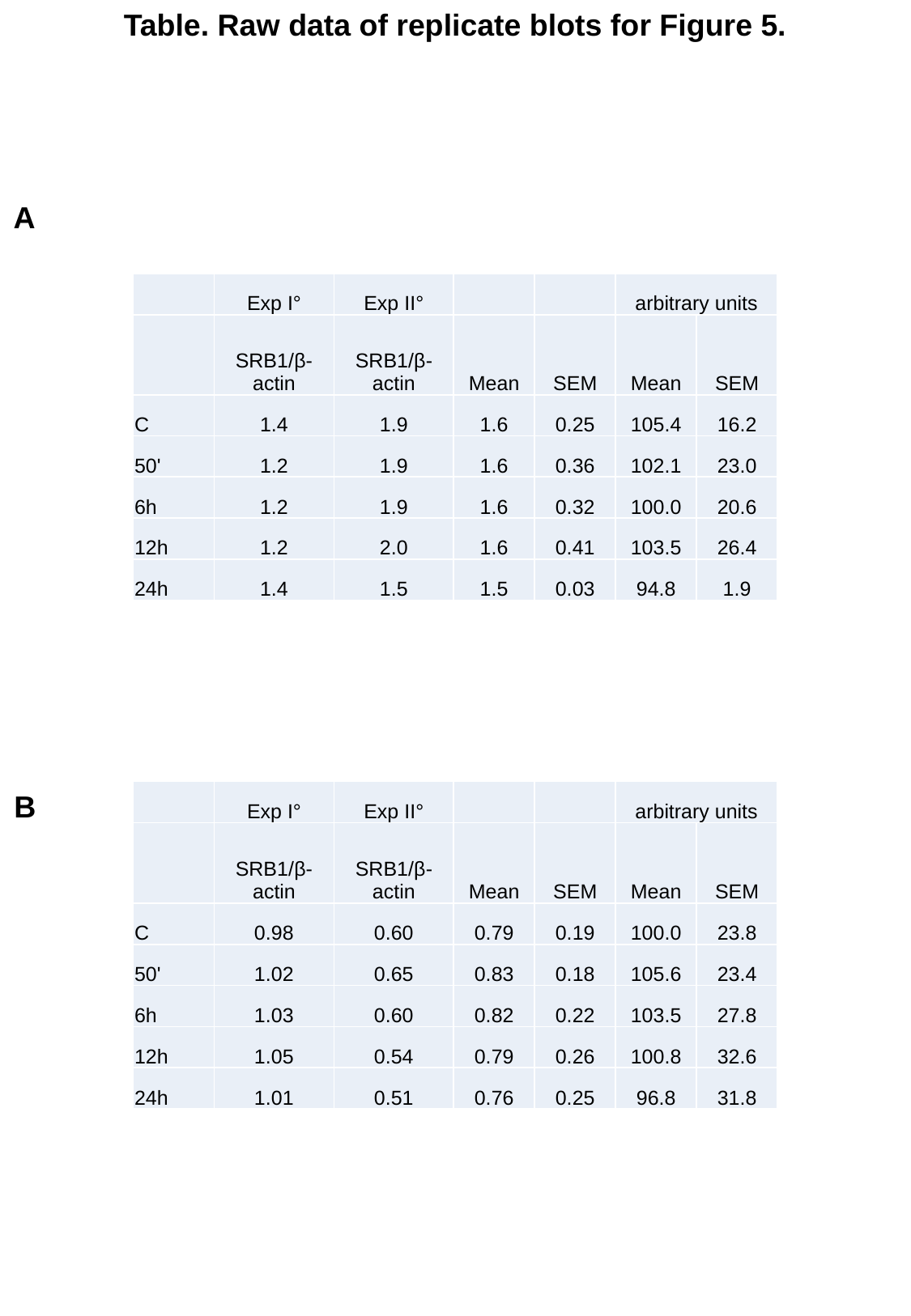

Table. Raw data of replicate blots for Figure 5.
A
| | Exp I° | Exp II° | | | arbitrary units | |
| --- | --- | --- | --- | --- | --- | --- |
| | SRB1/β-actin | SRB1/β-actin | Mean | SEM | Mean | SEM |
| C | 1.4 | 1.9 | 1.6 | 0.25 | 105.4 | 16.2 |
| 50' | 1.2 | 1.9 | 1.6 | 0.36 | 102.1 | 23.0 |
| 6h | 1.2 | 1.9 | 1.6 | 0.32 | 100.0 | 20.6 |
| 12h | 1.2 | 2.0 | 1.6 | 0.41 | 103.5 | 26.4 |
| 24h | 1.4 | 1.5 | 1.5 | 0.03 | 94.8 | 1.9 |
| | Exp I° | Exp II° | | | arbitrary units | |
| --- | --- | --- | --- | --- | --- | --- |
| | SRB1/β-actin | SRB1/β-actin | Mean | SEM | Mean | SEM |
| C | 0.98 | 0.60 | 0.79 | 0.19 | 100.0 | 23.8 |
| 50' | 1.02 | 0.65 | 0.83 | 0.18 | 105.6 | 23.4 |
| 6h | 1.03 | 0.60 | 0.82 | 0.22 | 103.5 | 27.8 |
| 12h | 1.05 | 0.54 | 0.79 | 0.26 | 100.8 | 32.6 |
| 24h | 1.01 | 0.51 | 0.76 | 0.25 | 96.8 | 31.8 |
B
