# Supplementary material for: Correction: Cigarette Smoke Affects Keratinocytes SRB1 Expression and Localization via H2O2 Production and HNE Protein Adducts Formation
Source: PLoS One. 2020 Jan 30;15(1):e0228663. doi: 10.1371/journal.pone.0228663 (PMC6992178; doi:10.1371/journal.pone.0228663)
Supplement: S2 File — In Exp II° data for Figure 5B, the membrane was cut to enable probing with two antibodies and the SRB1 western blot resulted in high molecular weight non-specific signals. The lower band in this SRB1 blot is the correct size for the SRB1 protein. (PPTX) [file pone.0228663.s002.pptx]

## Slide 1
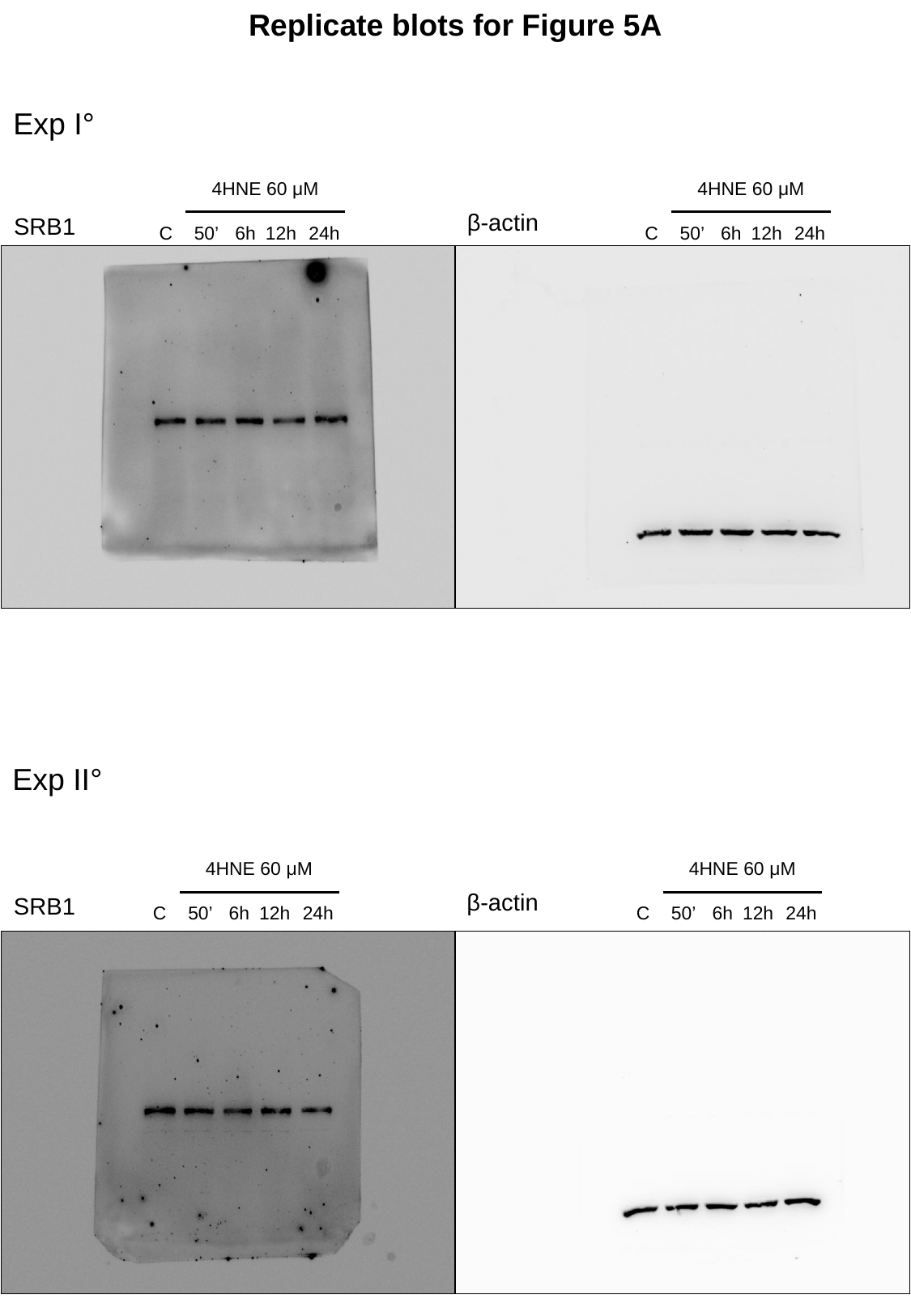

Replicate blots for Figure 5A
Exp I°
4HNE 60 μM
C
50’
6h
12h
24h
4HNE 60 μM
C
50’
6h
12h
24h
β-actin
SRB1
Exp II°
4HNE 60 μM
C
50’
6h
12h
24h
4HNE 60 μM
C
50’
6h
12h
24h
β-actin
SRB1

## Slide 2
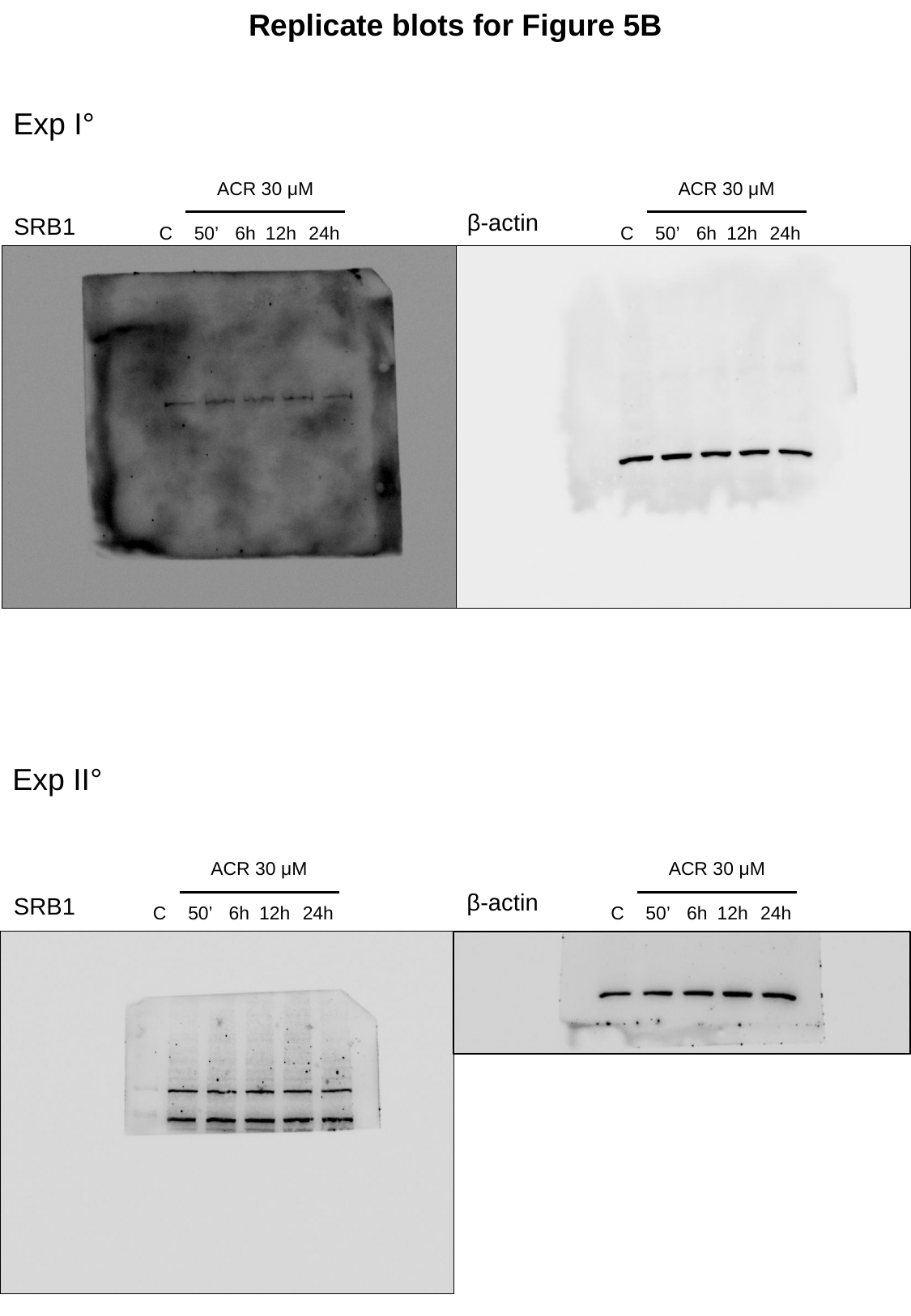

Replicate blots for Figure 5B
Exp I°
ACR 30 μM
C
50’
6h
12h
24h
ACR 30 μM
C
50’
6h
12h
24h
β-actin
SRB1
Exp II°
ACR 30 μM
C
50’
6h
12h
24h
ACR 30 μM
C
50’
6h
12h
24h
β-actin
SRB1
