# Supplementary material for: Correction: Cigarette Smoke Affects Keratinocytes SRB1 Expression and Localization via H2O2 Production and HNE Protein Adducts Formation
Source: PLoS One. 2020 Jan 30;15(1):e0228663. doi: 10.1371/journal.pone.0228663 (PMC6992178; doi:10.1371/journal.pone.0228663)
Supplement: S3 File — Note that the SRB1 blot on the final slide (labelled “5° exp. 12-01-2010”) includes a lower set of bands not present in other replicates of this experiment. The authors were unable to clarify the identity of these bands. (PPT) [file pone.0228663.s003.ppt]

## Slide 1
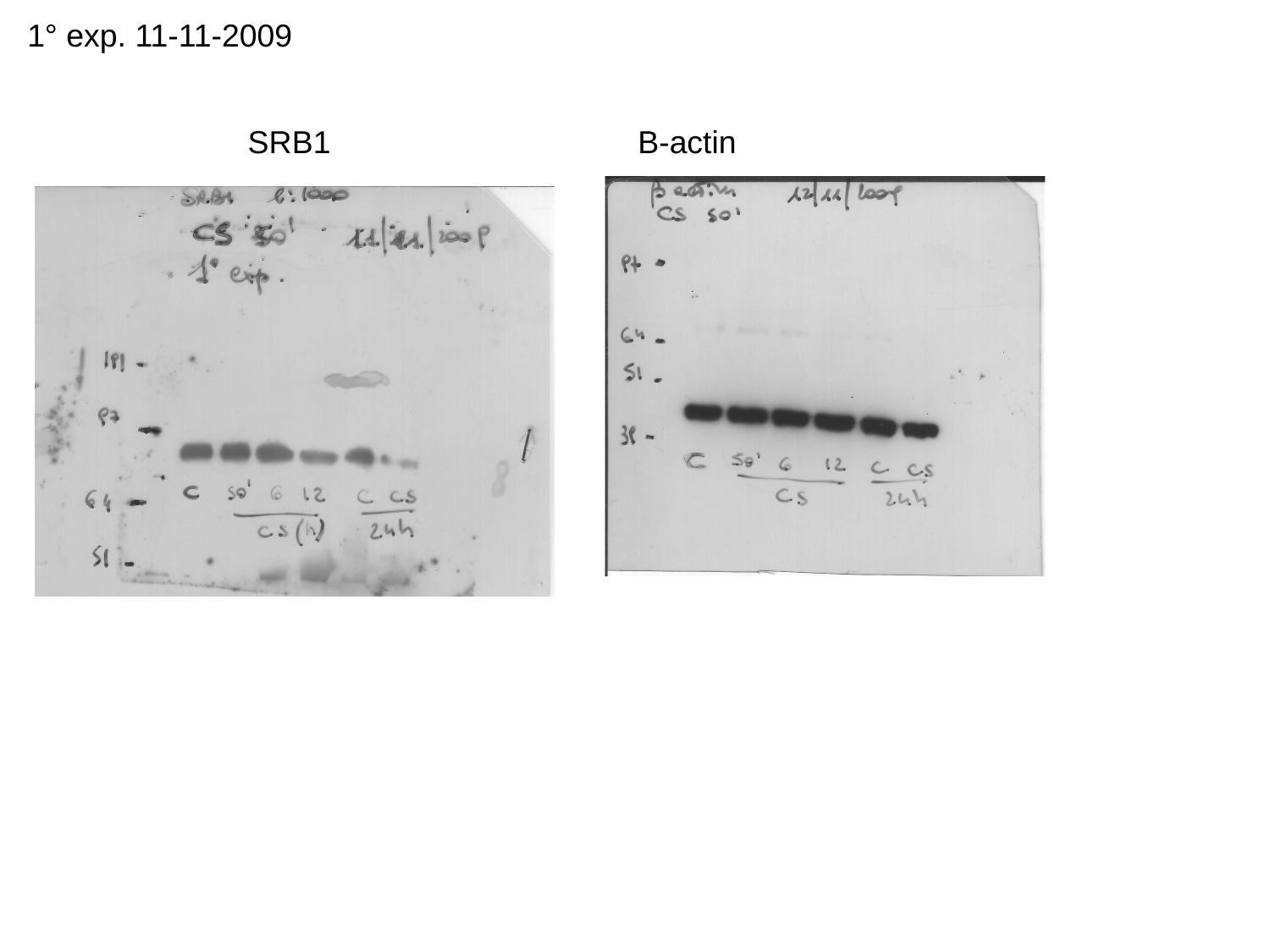

1° exp. 11-11-2009
SRB1
B-actin

## Slide 2
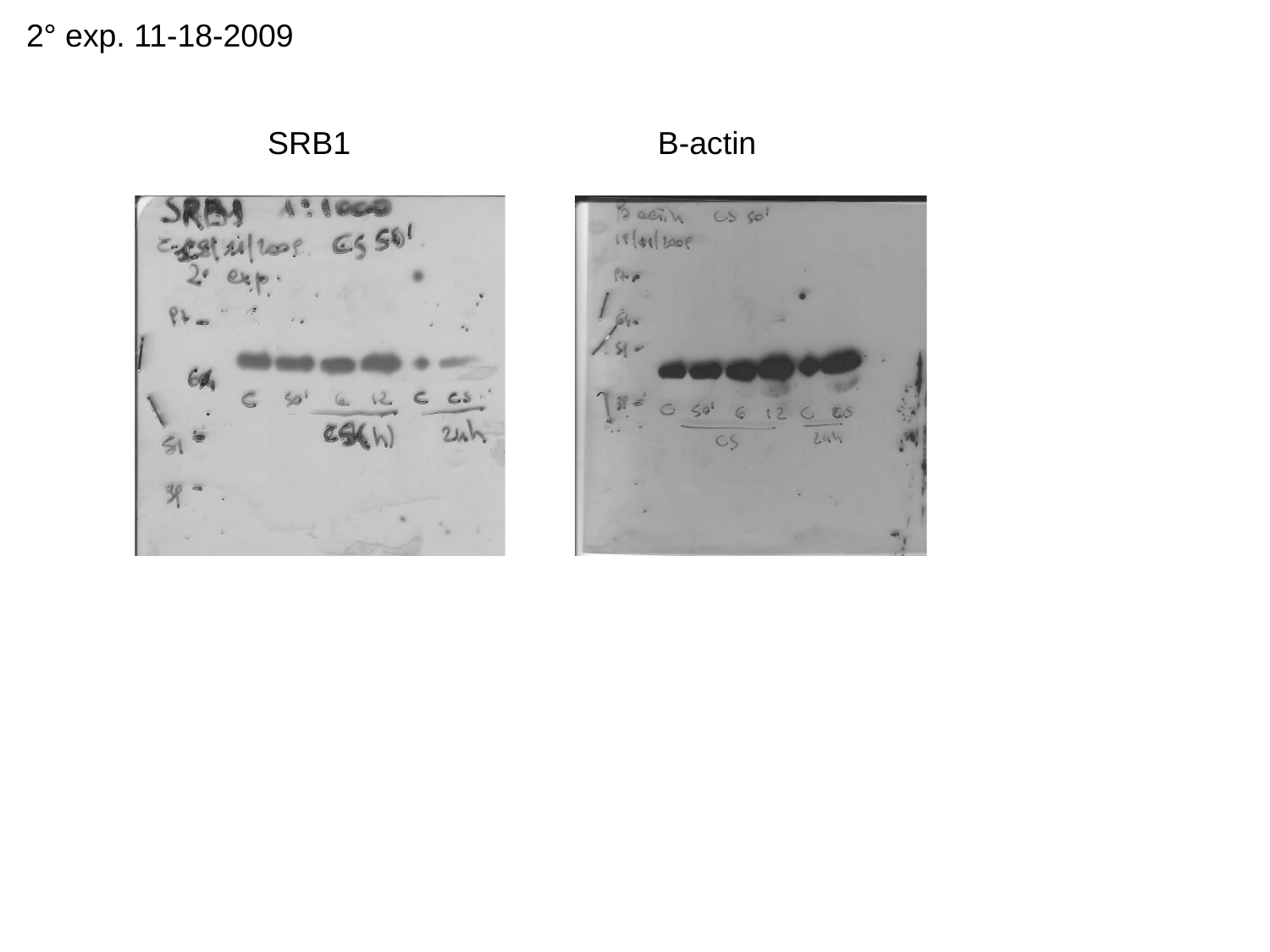

2° exp. 11-18-2009
SRB1
B-actin

## Slide 3
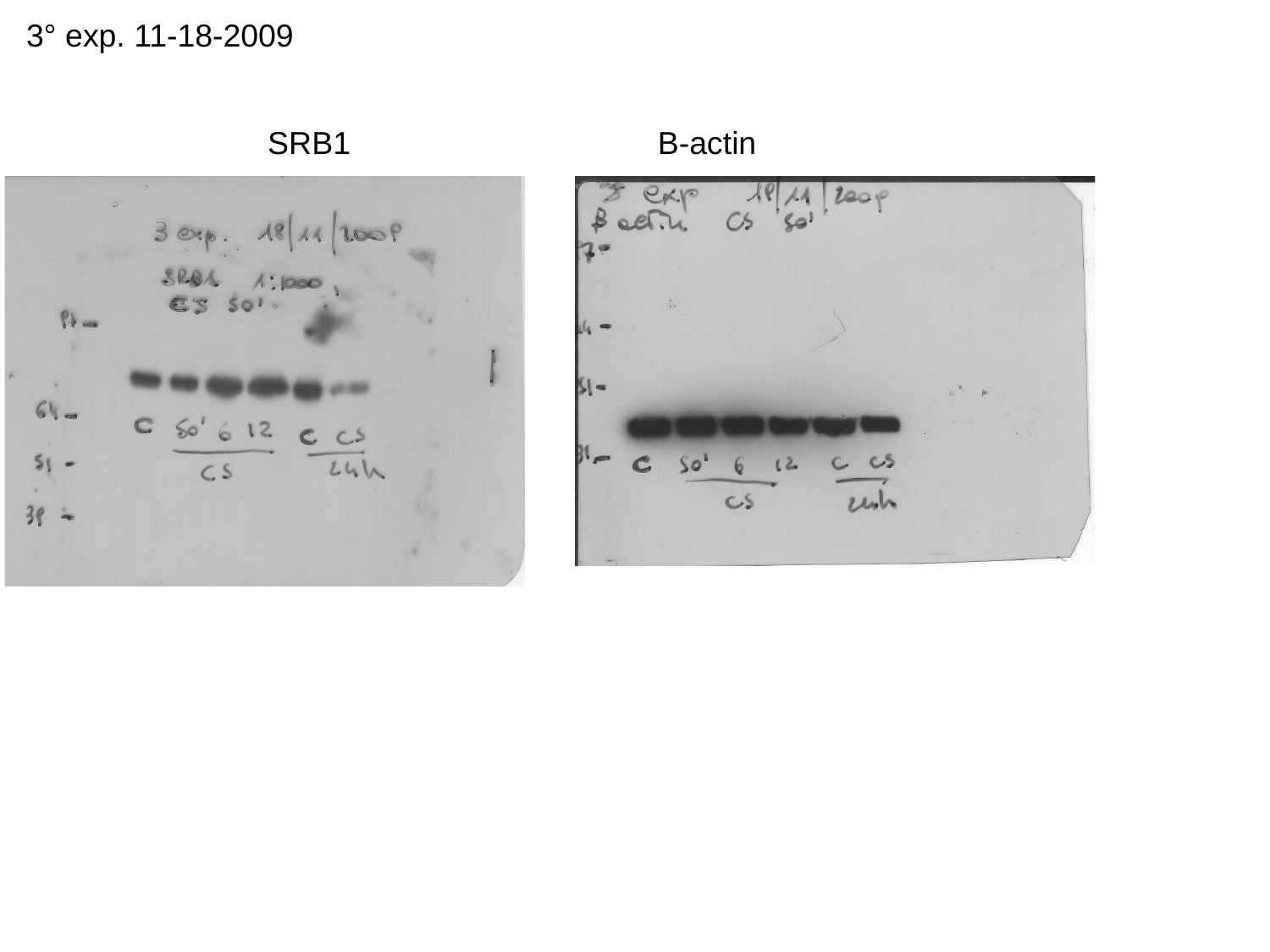

3° exp. 11-18-2009
SRB1
B-actin

## Slide 4
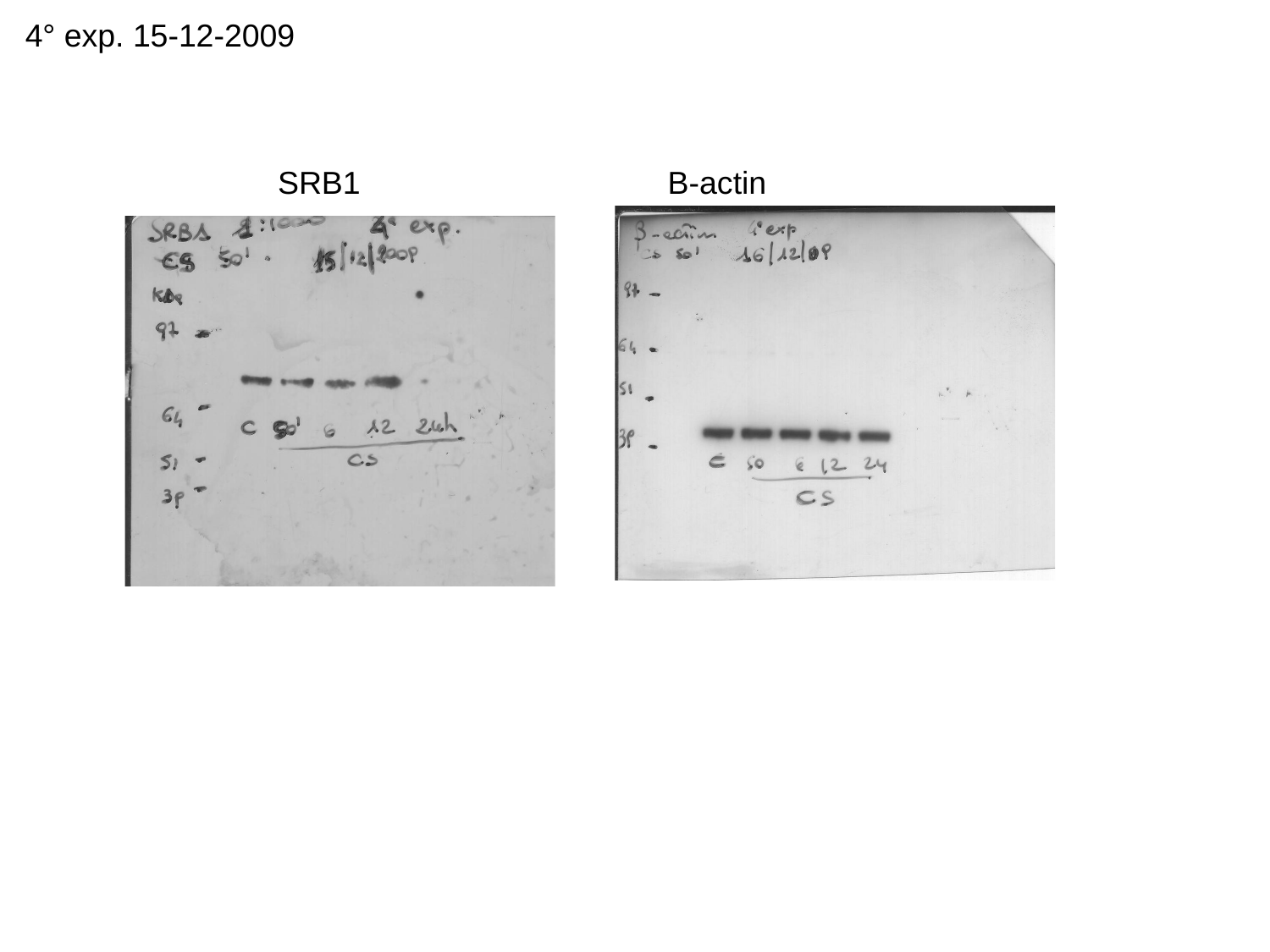

4° exp. 15-12-2009
SRB1
B-actin

## Slide 5
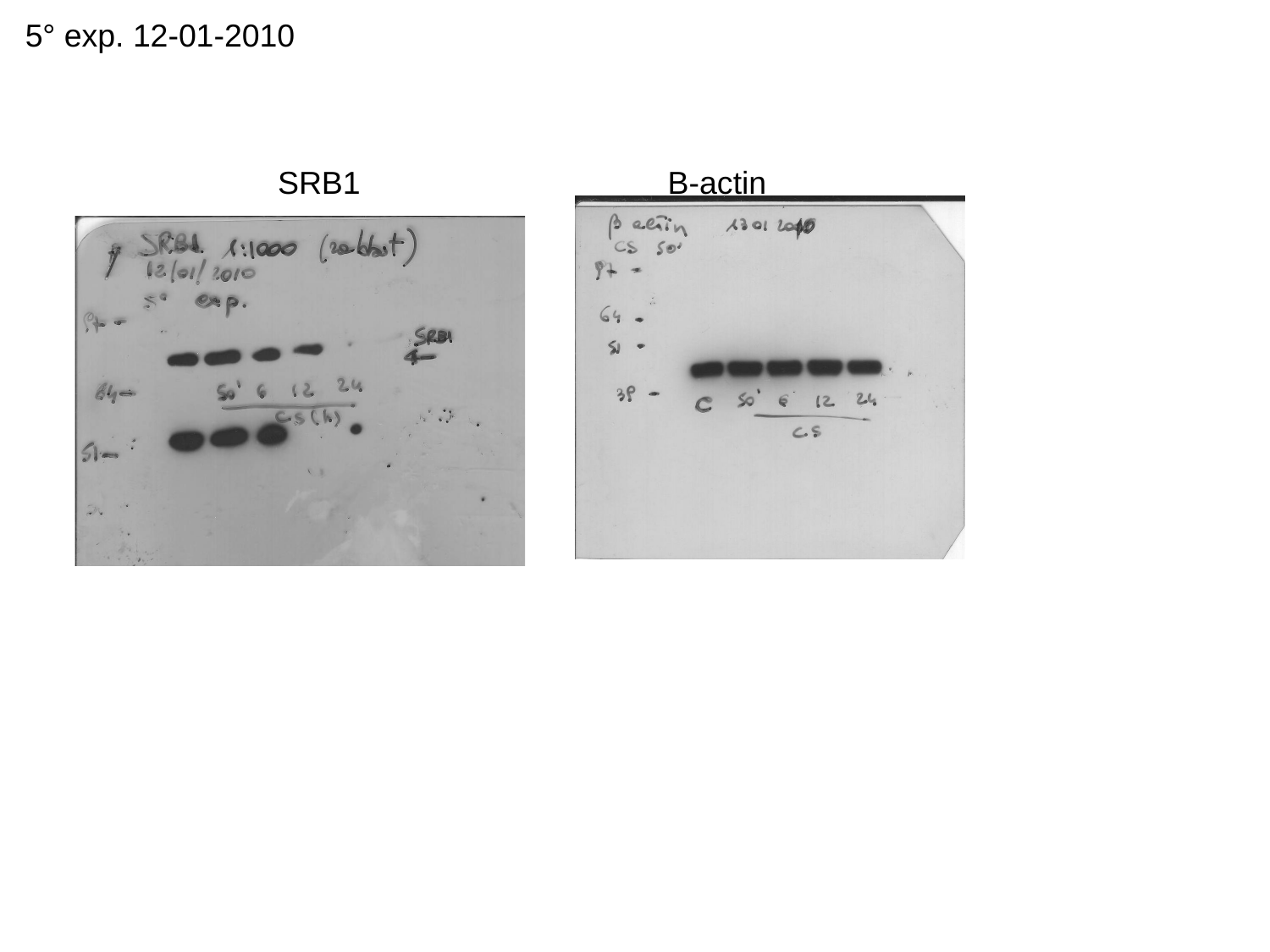

5° exp. 12-01-2010
SRB1
B-actin
